# Anion‐Blocking and Multipath‐Conducting Interfaces Enable Long‐Life Room‐Temperature Ester‐Based Ca‐Metal Batteries

**DOI:** 10.1002/advs.202512339

**Published:** 2025-09-12

**Authors:** Xuedong He, Jiarui Wang, Qingyang Cao, Yaohua Huang, Hongqing Li, Fei Tian, Huawei Song, Chengxin Wang

**Affiliations:** ^1^ State Key Laboratory of Optoelectronic Materials and Technologies School of Materials Science and Engineering Sun Yat‐Sen (Zhongshan) University Guangzhou 510275 P. R. China

**Keywords:** anion corrosion, Ca‐metal, ester electrolyte, interface kinetics, interphase regulation

## Abstract

Ester‐based lithium storage has been reliably validated for tens of years. However, calcium‐metal (Ca‐metal) batteries, as one of the most promising alternatives to lithium ion batteries, are restrained to several non‐ester or mixed‐cations electrolytes, while those utilizing pure calcium salt ester electrolytes have so far been considered impossible to durably work at room temperature due to easily passivated Ca‐metal anodes by anion corrosion, urgently necessitating stable and fast kinetics interfaces. Paired with a biomass‐derived carbon cathode, this work demonstrated the first case of long‐life pure calcium salt ester‐based Ca‐metal batteries of >500 cycles and 85.4% capacity retention at 25 mA g^−1^ by engineered Ca‐metal interfaces. Notably, the Ca‐metal electrodes also achieved 0.53 V (vs Ca/Ca^2+^) deposition overpotential, 6 mAh cm^−2^ interface‐stable deposit capacity, and >950 h deposition/stripping stability at 0.02 mA cm^−2^ and 0.02 mAh cm^−2^. The enhanced interface kinetics and reversibility is attributed to the engineered Ca‐metal interfaces with not only diverse interphases of calcium‐(Ca‐)/iron‐(Fe‐)based inorganic salts and core‐shell iron (Fe) nanocrystals affording abundant multiple ion/electron transportation pathways, but also compact carbon‐nitrogen (C─N organics as an effective anion‐blocking medium. This work opens an interface engineering avenue for ester‐based Ca‐metal batteries.

## Graphical abstract

Long‐life pure calcium salt ester‐based Ca‐metal batteries of >500 cycles and 85.4% capacity retention at 25 mA g^−1^ are demonstrated by the Ca‐metal anodes with engineered interfaces, which featured not only diverse interphases of Ca‐/Fe‐based inorganic salts and core‐shell Fe nanocrystals affording abundant multiple ion/electron transportation pathways, but also compact C─N organics as an effective anion‐blocking medium.

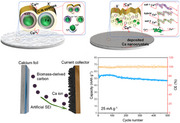

## 1 Introduction

The development of reliable rechargeable battery technologies is of great significance for sustainable development. Among various rechargeable batteries, ester‐based electrolytes have won from a series of electrolyte systems in the form of lithium ion batteries, and continue to be popular for decades, highly relying on their excellent comprehensive performance, including a series of indicates of ion conductance, viscosity, physical and chemical stability, wide voltage window, interface stability, and so on.^[^ ^1^ ^]^ However, due to performance bottlenecks, limited resources, and cost concerns of lithium‐based batteries, the development of other alternative or complementary rechargeable battery technologies, especially those compatible with ester electrolytes, has become an urgent need.^[^ ^2^ ^]^

Multivalent metals with high specific capacity and density offer significant advantages in terms of energy density, making the corresponding batteries as promising candidates.^[^ ^3^ ^]^ Among various multivalent metals, Ca stands out due to low redox potentials and widespread calcium resources availability in the Earth's crust.^[^ ^4^ ^]^ However, Ca still faces the issue of easy formation of an ionically insulating layer during Ca plating/stripping in common ester electrolytes at room temperature, which obstructs subsequent battery operation.^[^ ^5^ ^]^ Specifically, the spontaneously evolved solid electrolyte interphases (SEIs) cannot effectively avoid the unfavorable corrosion of Ca‐metal.^[^ ^5^, ^6^ ^]^ The corrosion process, especially that involved with the undesirable anionic reactions, easily leads to the evolution of electronically and ionically dual‐insulating barriers, hindering efficient charge/mass flows across the Ca‐electrolyte interfaces.^[^ ^7^ ^]^ Consequently, it is crucial to develop appropriate SEIs for Ca‐metal electrodes with fast Ca^2+^ transport kinetics, so as to realize stable and reversible plating/stripping processes in common electrolytes.^[^ ^5^, ^8^ ^]^

The last few years have witnessed several strategies to achieve reversible Ca electrodes at room temperature, such as stabilizing Ca ions with weakly coordinating anions and designing reversible Ca plating/stripping guided by high donor number solvents.^[^ ^9^ ^]^ However, they were generally confined to the optimization of electrolytes through utilizing non‐ester solvents or the incorporation of Li/Na/K‐based salts to construct hybrid‐ion SEIs.^[^ ^9^, ^10^ ^]^ These approaches have alleviated the issues of unfavorable SEIs formation from different aspects, thereby largely enhancing the Ca reaction kinetics. Even so, the long‐term cyclability and reversibility are still challenged by the issues regarding the interfacial stability.^[^ ^6^, ^11^ ^]^ Ca‐metal electrodes reversibly and stably operating in pure calcium salt ester electrolytes, such as calcium trifluoromethylsulfonylimide (Ca(TFSI)_2_), calcium perchlorate (Ca(ClO_4_)_2_), and calcium tetrafluoroborate (Ca(BF_4_)_2_), for long‐term ester‐based batteries, are still unachievable. As we know, ester‐based solvents are well compatible with existing Li‐ion battery technology, thus facilitating potential commercialization processes subsequently. Besides, relevant researches also provide valuable insights into SEIs or interfaces for efficient ion storage.^[^ ^12^ ^]^ Although N‐rich interphases in SEIs significantly accelerated Ca reaction kinetics in ester‐based electrolytes, enabling reversible plating/stripping cycles for hundreds of hours, the surface of Ca electrodes still tended to be passivated rapidly as the current rates increased, showing sharply ascending polarization potentials.^[^ ^7c^ ^]^ Consequently, the design and development of interfaces that simultaneously promote rapid Ca^2^⁺ diffusion and ensure stable Ca plating/stripping remains a formidable challenge, particularly in the context of ester‐based electrolytes.

**Scheme 1:**
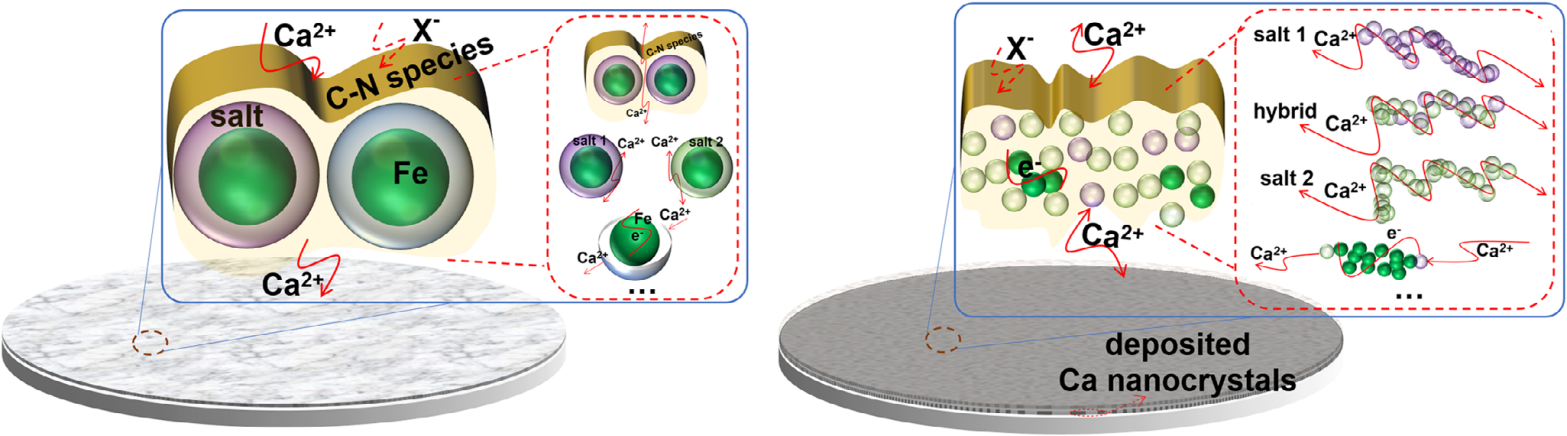
Illustration of anion‐blocking and multipath‐conducting interfaces at the Ca‐metal electrodes before (left) and after (right) cycling.

In this study, a reactive ion‐exchange strategy was introduced to construct artificial interphases featuring both anion‐blocking and multipath‐conducting interfaces for Ca‐metal anodes. Through controlled chemical reactions between Ca‐metal and ferrous chloride (FeCl_2_), as well as N, N‐dimethylformamide (DMF), the constructed interphase layer integrates at least three types of functional components: (i) ion‐transport channels composed of Ca‐/Fe‐based inorganic salts, (ii) electron‐conductive pathways established by core‐shell Fe nanocrystals, and (iii) anion‐blocking domains formed by N‐rich organic species as illustrated in the left panel of **Scheme** **1**. Furthermore, extremely pulverized interphase species after cycling availed the evolution of N‐containing compact SEIs, efficiently shielding the invasion of corrosive anions, and meanwhile maintained the favorable multipath conduction effect as shown in the right panel of Scheme 1. As a result, both enhanced interface kinetics and reversibility were simultaneously achieved for the Ca electrodes with the artificial interphase layer in 0.5 m Ca(BF_4_)_2_ propylene carbonate/dimethyl carbonate (PC/DMC, v/v = 1:1) electrolyte, demonstrating stable plating/stripping processes for 950 h at 0.02 mA cm^−2^ without any significant potential drift. The interfaces before and after plating/stripping reveal that extremely pulverized amorphous species of Fe‐/Ca‐salts, and C─N organics densely filled in the SEIs effectively protect Ca metal anodes from anion corrosion, meanwhile provide abundant interface pathways for rapid Ca^2^⁺ diffusion.

## 2 Results and Discussion

### 2.1 Artificial Interphases of the Ca‐Metal Anodes

**Figure 1:**
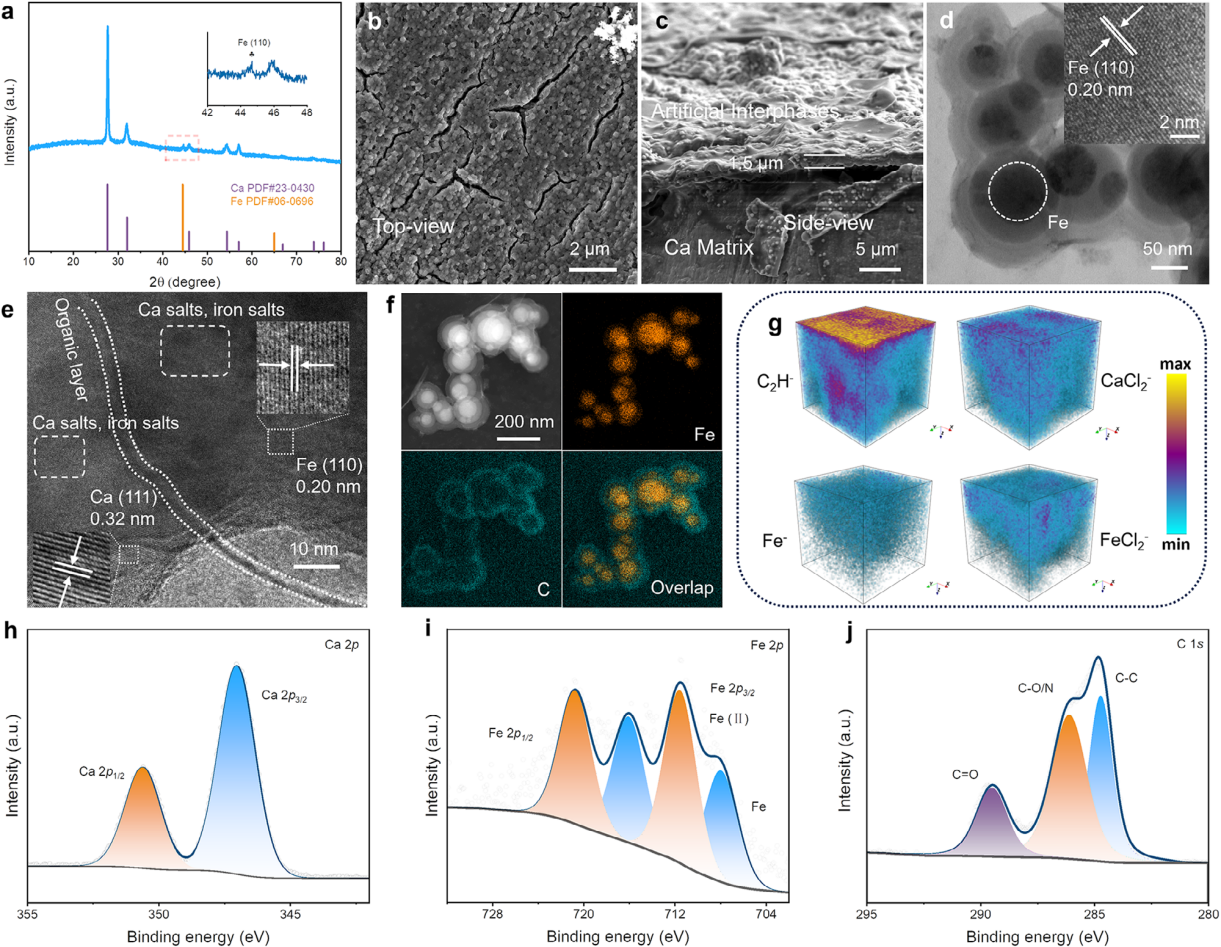
Interphase characterization of FeCl_2_‐treated Ca electrodes before cycling. a) XRD pattern, b,c) top‐ and side‐view SEM, d) TEM, e) HRTEM, f) STEM, and corresponding EDS mapping. g) TOF‐SIMS 3D render overlay images. High‐resolution h) Ca 2p, i) Fe 2p, and j) C 1s XPS spectra.

The interphase layer was evolved by a reactive ion‐exchange process between fresh Ca foil (named pristine Ca) and drop‐casting 0.5 m FeCl_2_ DMF solution. Owing to the substantial difference in reduction potentials between Ca and Fe, the reaction Ca + FeCl_2_ → CaCl_2_ + Fe spontaneously occurs in the organic solution. Simultaneously, complex chemical reactions between the DMF solution and Ca generate significant amorphous products as previously reported.^[^ ^7c^ ^]^ The X‐ray diffraction (XRD) pattern of the Ca‐metal after reaction (named FeCl_2_‐treated Ca, **Figure** **1**a) displays several remarkable reflection peaks at 27.6°, 32.0°, 45.9°, 54.4°, and 57.0°, well consistent to cubic Ca (PDF No. 23–0430). Besides, a small and broadening peak at 44.5° ascribed to cubic Fe (PDF No. 06–0696), suggests that part of the Fe^2+^ ions were reduced into nanocrystals of elemental Fe by superficial Ca‐metal. In the meanwhile, superficial Ca‐metal itself would be spontaneously transformed into Ca salts with very low crystallinity, as no corresponding diffraction peaks were observed, except for the broadened amorphous package ≈22.5°. Furthermore, the residual Fe^2+^ ions and DMF molecules contributed to the formation of various amorphous species, e.g., the poorly crystallized Ca‐/Fe‐based inorganic salts and C─N organics.^[^ ^7^, ^10^ ^]^

The scanning electron microscopy (SEM) images (Figure 1b,c) show that the interphase layer appears as a uniform coating of ≈1.5 µm, consisting of compactly packed spherical nanoparticles with diameters of 100–200 nm. The microcracks, occasionally appearing in the upper surface, were possibly caused by the densification effect of the evacuation procedure in the SEM characterization, similar to those observed in tape‐casting electrodes.^[^ ^13^ ^]^ The SEM energy dispersive X‐ray spectroscopy (EDS) element mapping images (Figure S1, Supporting Information) exhibit that C, N, Ca, Fe, and Cl elements are obviously gathering in the coating. The spherical nanoparticles encapsulated in the coating are further confirmed as core‐shell structures ≈100–200 nm in the transmission electron microscopy (TEM) image (Figure 1d). The cores ≈60–70 nm present well‐defined lattice fringes with the interplane spacing of 0.2 nm (inset view of Figure 1d), consistent with cubic Fe. The shells exhibit an amorphous character in the high‐resolution TEM (HRTEM, Figure 1e) image, and are possibly composed of salts of Ca and residual FeCl_2_ with very poor crystallinity and some C─N organic species. The distinct disparities in contrast and element distribution of the cores and shells are further verified in the high‐angle annular dark field‐scanning TEM (HAADF‐STEM) and corresponding EDS mapping images (Figure 1f). The formation of this distinct structure may be attributed to the varied diffusion kinetics of various ions.^[^ ^14^ ^]^

To confirm the amorphous salts and N‐containing organics, the interphase layer was further characterized by time‐of‐flight secondary ion mass spectrometry (TOF‐SIMS), Fourier‐transform infrared (FT‐IR) spectrum, and X‐ray photoelectron spectroscopy (XPS). The 3D render overlay images of TOF‐SIMS depth profiling in Figure 1g show remarkable secondary ion signals of C_2_H^−^, Fe^−^, CaCl_2_^−^, and FeCl_2_^−^, implying abundant salts and organics in the interphase layer. The C_2_H^−^ fragments originate from the organics, while Fe^−^, FeCl_2_^−^, and CaCl_2_^−^ species come from Fe nanocrystals and the salts. The more pronounced signal of C_3_^−^ at the outer layer than that for the inner layer, together with the initial intensified and then weakened signal of Fe^−^ (Figure S2, Supporting Information), indirectly proves the encapsulation structure of Fe (for elemental Fe nanocrystals or amorphous Fe salt) by organic species. FT‐IR spectrum (Figure S3, Supporting Information) substantiates that the organic species contain abundant organic functional groups as verified by the strong absorbance bands of 1258 cm^−1^ (1058 cm^−1^), 1655 cm^−1^, 1507 cm^−1^ (1099 cm^−1^), 1386 cm^−1^ corresponding to vibrations of C─O, C═O, C─C, and C─N respectively. In contrast to C─N organic species previously reported from the Ca‐DMF reaction, abundant amorphous salts were also formed in situ in the interphase layer.^[^ ^7c^ ^]^ Moreover, XPS survey spectrum confirms the existence of C, N, O, Ca, Fe, and Cl elements in the interphases (Figure S4a, Supporting Information), in good accordance with the SEM and TEM results. High‐resolution Ca 2p XPS spectrum with binding energies at 347.05 eV (2p_3/2_), and 350.7 eV (2p_1/2_) for Ca salt and Fe 2p XPS spectrum with deconvoluted peaks at 708.3 (2p_3/2_) and 716.3 eV (2p_1/2_) for Fe^0^, 711.8 (2p_3/2_) and 720.8 eV (2p_1/2_) for Fe^2+^ indicate the existence of Ca‐/Fe‐based salts and elemental Fe in the interphases.^[^ ^15^ ^]^ C─N organic species are also verified in the C 1s and N 1s XPS spectra (Figure 1j; Figure S4b, Supporting Information) with deconvoluted peaks at 284.4, 285.95, 288.15, and 289.45 eV for C─C, C─O, C─N, and C═O bonding, respectively.^[^ ^16^ ^]^ The Cl 2p_1/2_ (194.4 eV), and 2p_3/2_ (199.1 eV) signals in Cl 2p XPS spectrum reveal the existence of Cl element in the Ca‐/Fe‐based salts (Figure S4c, Supporting Information).^[^ ^17^ ^]^

Based on the above result, the interphase layer consists of abundant Fe nanocrystals, Fe‐/Ca‐based amorphous salts, and amorphous C─N organic species. Among them, the core‐shell structured Fe and various interfaces will largely favor for enhanced ion/electron diffusion kinetics as illustrated in Scheme 1, while compactly filling C─N organic species function as an anion‐blocking buffer layer to suppress the anion corrosion. Simultaneously, calcium‐ion conductivity can also be enhanced by chlorine‐containing compounds for lowering barriers for Ca^2+^ interstitial hopping and high polarizability of Cl^−^ softening lattice structures.^[^ ^18^ ^]^ Therefore, enhanced interface kinetics and reversibility can be expected for the as‐prepared Ca‐metal anodes.

### 2.2 Kinetics and Reversibility of the Ca‐Metal Anodes

**Figure 2:**
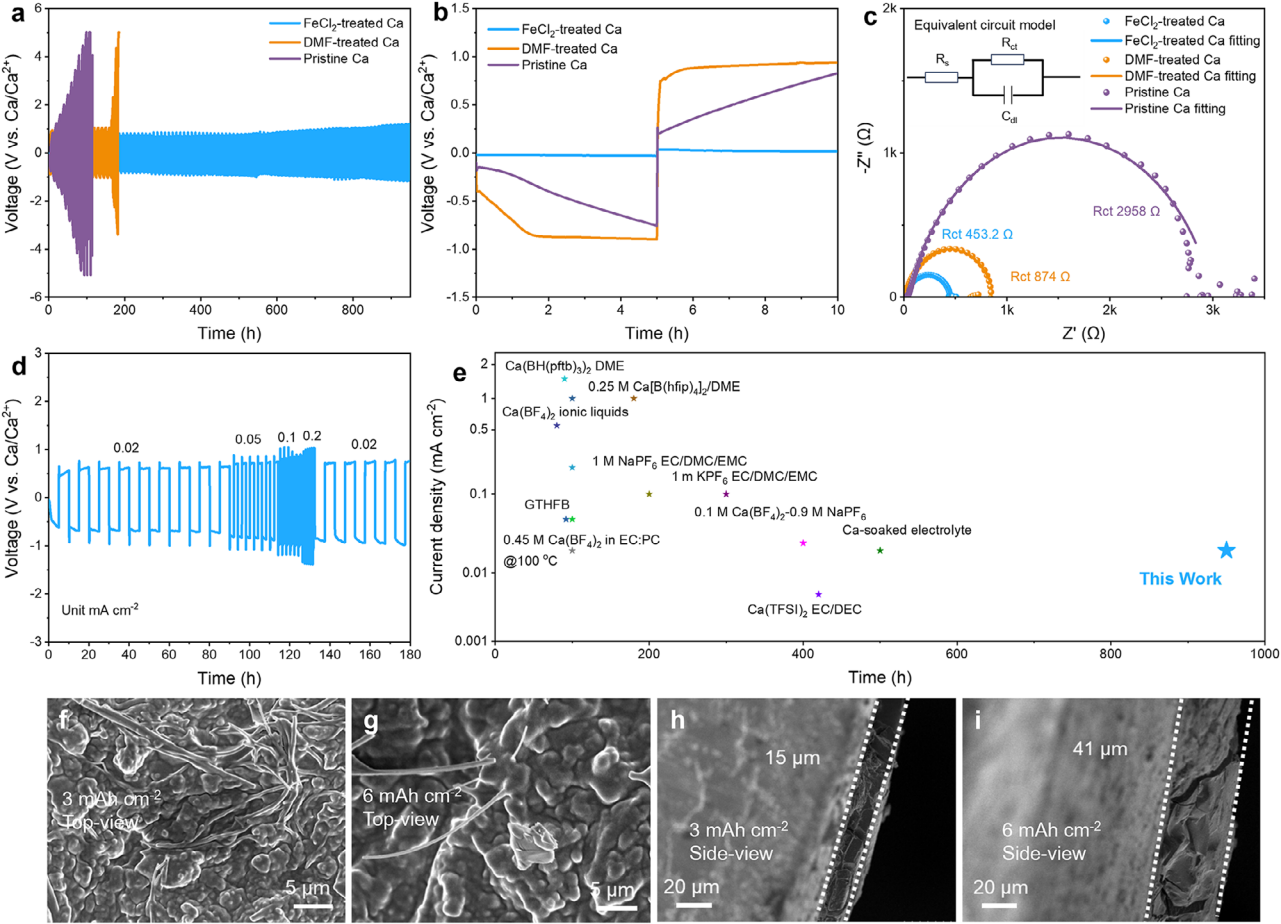
The electrochemical characterization of different Ca‐metal electrodes. Voltage‐time profiles at 0.02 mA cm^−2^ for a) cycling stability and b) initial cycle. c) EIS. d) Voltage–time profiles for rate performance at varying rates from 0.02 to 0.2 mA cm^−2^. e) Cycling performance comparison among Ca‐metal anodes in this work and others based on the data documented in Table S1 (Supporting Information). Deposition microstructure stability of FeCl_2_‐treated Ca electrodes at f,h) 3 mAh cm^−^^2^ and g,i) 6 mAh cm^−^^2^.

To evaluate the effectiveness of the interphase layer, calcium tetrafluoroborate in a propylene carbonate/dimethyl carbonate mixed solvent (0.5 m Ca(BF_4_)_2_ PC/DMC), which was previously confirmed to be unfavorable for reversible Ca plating/stripping at room temperature, was used as the electrolyte.^[^ ^19^ ^]^ The plating/stripping reversibility was examined by galvanostatic charge‐discharge cycling at 0.02 mA cm^−2^ and 1 h for each cycle. As shown in **Figure** **2**a, the voltage‐time profiles of pristine Ca electrodes rapidly rise to 5 V (vs Ca/Ca^2^⁺) within 100 h. The severe polarization phenomenon indicates that pristine Ca electrodes suffer persistent passivation and corrosion due to the formation of insulating interphases. This observation aligns with the findings reported for other fluorinated Ca salt ester electrolytes.^[^ ^5^, ^7^ ^]^ Differently, the fresh Ca foil pretreated with pure DMF (named DMF‐treated Ca for comparison) exhibits enhanced plating/stripping reversibility, stably maintaining for 165 h with a polarization potential of ≈1 V (vs Ca/Ca^2^⁺), comparable to that of the Ca‐metal electrodes modified with a compact N‐rich interphase layer.^[^ ^7c^ ^]^ In contrast, the initial polarization potential of FeCl_2_‐treated Ca electrodes (Figure 2b) appears at 0.04 V (vs Ca/Ca^2+^) at the first plating/stripping cycle, and then slowly rises up to ≈0.9 V (vs Ca/Ca^2+^) after initial cycling. The lowered polarization lasts for more than 950 h with very little potential drift. The corresponding reversible deposition potential was further confirmed as −0.53 V (vs Ca/Ca^2+^) in Swagelok‐type three‐electrode tests with Ag/Ag^+^ electrode calibrated by ferrocene/ferrocenium (Fc/Fc^+^) redox couple as the reference (Figure S5, Supporting Information), evidently higher than that of ‐1 V (vs Ca/Ca^2+^) for the DMF‐treated Ca electrode. The reduced deposition overpotential and stable cycling demonstrate that the interphase layer significantly enhances the kinetics and reversibility of FeCl_2_‐treated Ca electrodes. While this low‐current validation establishes the interphase's functionality, future work will explore high‐current regimes (>0.5 mA cm^−2^) to assess practical relevance. Nevertheless, the present achievement of stable cycling in ester‐based electrolytes represents a fundamentally important breakthrough.

The enhanced kinetics and reversibility are also evidenced by the electrochemical impedance spectroscopy (EIS), cyclic voltammetry (CV), Tafel, and the microstructure evolution of the deposition layer. The equivalent circuit in Figure 2c (inset) enables direct analysis of the variation in charge transfer resistance (R_ct_), as indicated by the semicircular diameter in the Nyquist plots (Figure 2c).^[^ ^20^ ^]^ FeCl_2_‐treated Ca and DMF‐treated Ca electrodes deliver R_ct_ of only 453.2 and 874 Ω, significantly lower than that of 2958 Ω for pristine Ca electrodes. The substantially reduced R_ct_ value for the charge‐transfer processes indicates that artificial interphase layers notably enhance the calcium reaction kinetics. The CV curves (Figure S6, Supporting Information) display that a considerable peak current of 0.22 mA cm^−2^ is attained at 1 mV s^−1^ for FeCl_2_‐treated Ca electrodes, much larger than those of DMF‐treated Ca and pristine Ca electrodes (0.089 and 0.051 mA cm^−2^), indicating remarkably improved reversibility and reaction kinetics for Ca plating/stripping processes. The Tafel plots (Figure S7, Supporting Information) demonstrate that the dissolution potential of FeCl_2_‐treated Ca was 0.086 V lower than that of pristine Ca, indicating reduced passivation due to the protective interphase layer. The change of Ca‐ion transference number from merely 0.025 of pristine Ca electrode to 0.1 of FeCl_2_‐treated Ca electrode (Figure S8, Supporting Information) also confirms the enhanced transfer kinetics of Ca^2+^ and suppressed penetration of fluorinated anions. The above analyses demonstrate that the interphase layer is efficient in lowering interfacial barriers and accelerating Ca^2+^ transport kinetics. Therefore, excellent rate performance (Figure 2d) is also achieved for FeCl_2_‐treated Ca electrodes, in addition to the good reversibility comparable to some of the best results reported (Figure 2e; Table S1, Supporting Information). Moreover, microstructures of FeCl_2_‐treated Ca electrodes (Figure 2f–i) after plating at large capacities of 3 and 6 mAh cm^−2^ remain smooth and dense, in stark contrast to the porous and loose architecture observed in pristine Ca and DMF‐treated Ca electrodes (Figure S9, Supporting Information). Thickness measurements of Ca deposited at different capacities showed that 3 mAh cm^−2^ yields a thickness of 15 µm, while 6 mAh cm^−2^ results in 41 µm. According to Faraday's law, the theoretical thickness for 1 mAh cm^−2^ of electrodeposited Ca is 4.8 µm. The close agreement between experimental and theoretical values suggests minimal deviations, primarily attributable to factors such as lower Coulombic efficiency, SEI consumption, and substrate roughness. Ca deposition on a Li foil was also performed to exclude potential interference from the Ca substrate. A distinct diffraction peak at 27.6° corresponding to the Ca (111) plane was observed (Figure S10, Supporting Information), confirming successful Ca plating on the Li substrate. The corresponding SEM/EDS element mapping images (Figures S11 and S12, Supporting Information) visually demonstrate the successful electroplating of Ca onto the Li foil by the distinct element signals of Fe and Ca on the surface of the Li foil. A series of plating/stripping tests (Figures S13–S18, Supporting Information) of Ca‐metal pretreated by other metal chlorides also demonstrates the good effect of the unique interphase layer on the cycling stability, even outperforming some popular ether electrolytes.

### 2.3 SEIs of Ca‐Metal Anodes

**Figure 3:**
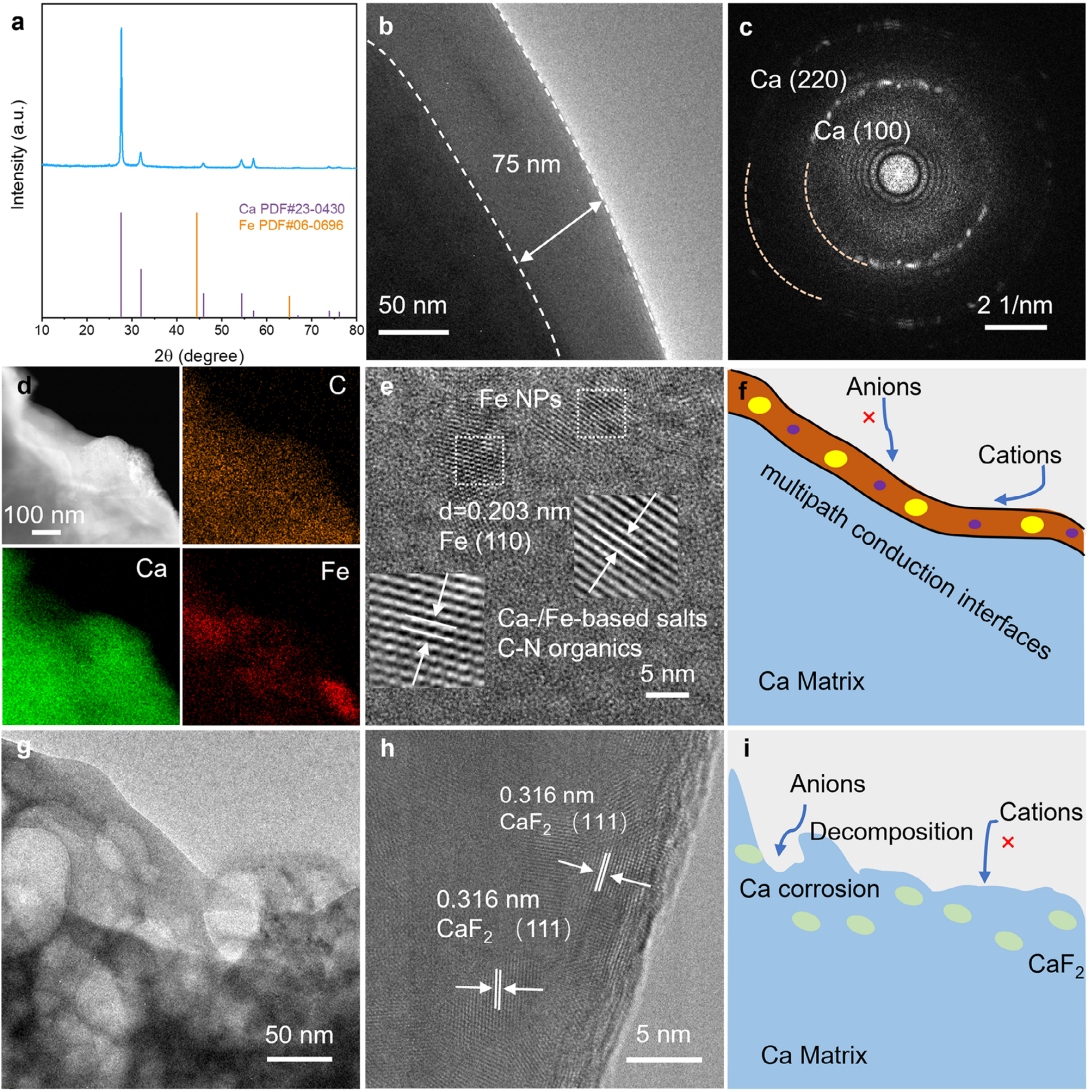
Interphase characterization of FeCl_2_‐treated Ca in comparison with pristine Ca electrodes after cycling. a) XRD, and b) TEM, c) SAED, d) HAADF‐STEM and corresponding EDS mapping, and e) HRTEM images, and f) SEI schematic of FeCl_2_‐treated Ca electrodes. g) TEM and h) HRTEM images, and i) SEI schematic of pristine Ca electrodes.

The kinetics and reversibility are also highly relevant to SEIs. The XRD pattern of the cycled FeCl_2_‐treated Ca (**Figure** **3**a) indicates that large Fe nanocrystals in the interphase layer suffered from severe pulverization during cycling, verified by the disappearance of the diffraction peak at 44.5° for cubic Fe. The TEM image (Figure 3b) reveals the extremely pulverized amorphous or poorly crystallized interphases as a homogeneous SEI layer of ≈75 nm. The selected area electron diffraction (SAED) image (Figure 3c) confirms its typical diffuse diffraction halo, like that of some amorphous materials, along with some scattered spots attributed to the diffraction of (100) and (220) crystal planes of deposited Ca nanocrystals. The HAADF‐STEM and EDS element mapping images (Figure 3d) substantiate uniformly distributed C elements from C─N organic species, and interconnected distributions of Fe and Ca elements from Fe ultra‐small nanocrystals and Fe‐/Ca‐based salts in the SEI layer. These species are further confirmed in the HRTEM image (Figure 3e) with compactly packed amorphous zones of C─N organic species and Ca‐/Fe‐based salts, and nanocrystals with an average size of ≈5 nm, showing an interplanar spacing of 0.203 nm attributed to (110) planes of Fe.

The SEI microstructure, composed of compactly packed Ca‐/Fe‐based salts, small Fe nanocrystals, and organic C─N species, provides abundant interfaces for rapid cation diffusion, meanwhile maintaining enough blocking effect for the penetrating of large corrosive anions (Figure 3f). Fragmented nanocrystals demonstrate superior Ca^2+^ ion conductivity, attributable to two key mechanisms. Thermodynamically, their high defect concentration reduces diffusion barriers, while kinetically, the interconnected grain boundary network establishes rapid transport routes. Additionally, mixed ionic‐electronic conduction pathways were also established—crystalline domains facilitating electronic transport through metallic Fe networks, and amorphous phases providing rapid ionic transport channels via calcium salts and C─N organic species. By contrast, the TEM image of the cycled pristine Ca (Figure 1g) exhibits a porous surface structure resulting from uncontrolled anion corrosion.^[^ ^6^ ^]^ The corresponding HRTEM image (Figure 1h) confirms the porous passivation layer with clear lattice fringes with a spacing of 0.316 nm, consistent with that of (111) crystal planes of cubic CaF_2_ (PDF No. 87–0971). A significantly higher F/Ca elemental ratio observed in SEM‐EDS (Figure S9e, Supporting Information) further confirms the accumulation of fluoride through continuous anion decomposition. With such uneven corrosion (Figure 1i), the pristine Ca electrode can hardly form an effective protective layer. Consequently, continuous anion decomposition leads to progressive accumulation of insulating byproducts.

**Figure 4:**
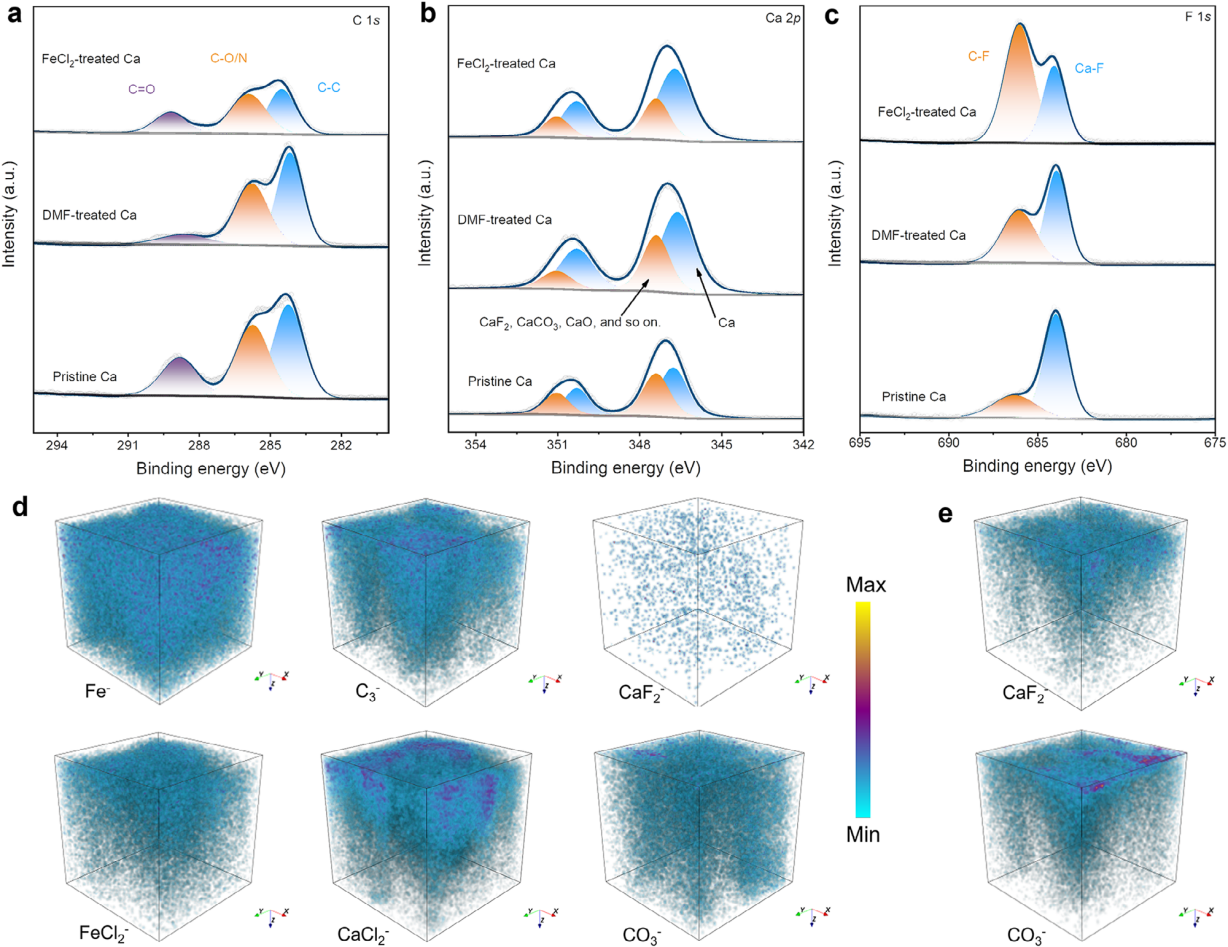
Deconvoluted local a) C 1s, b) Ca 2p, c) F 1s XPS spectra of different Ca‐metal electrodes after cycling. TOF‐SIMS 3D render overlayer images of d) FeCl_2_‐treated Ca and e) pristine Ca electrodes after cycling.

Moreover, XPS, TOF‐SIMS, and FT‐IR of the cycled Ca‐metal electrodes were also carried out to confirm the species in the SEI. The denoted C─C (284.8 eV), C─O/‐N (286.2 eV), and C═O (289.5 eV) bonding components in the deconvoluted C 1s XPS spectra (**Figure** **4**a) indicate the existence of C─N or C─O organic species or carbonates in the SEIs, which are from electrolyte decomposition or interphase‐electrolyte interactions.^[^ ^16^, ^21^ ^]^ The deconvoluted Ca 2p XPS spectra (Figure 4b) show two couples of split peaks of 346.5 eV (Ca 2p_3/2_) and 350.1 eV (Ca 2p_1/2_), and 347.5 eV (Ca 2p_3/2_) and 351.3 eV (Ca 2p_1/2_), ascribed to elemental Ca and Ca‐based compounds, respectively.^[^ ^22^ ^]^ The C‐F (686.4 eV) and Ca‐F (684.2 eV) signals in the deconvoluted F1s XPS spectra (Figure 4c) imply the existence of CaF_2_ and organic fluorine in the SEIs. Quantitative analysis of the spectral peaks shows that the SEI of pristine Ca is dominated by inorganic calcium salts such as CaF_2_ and CaCO_3_, while those of Ca‐metal with artificial interphase layers contain abundant organics, especially the introduction of C─N species (Figure S19, Supporting Information). Besides, some borate was also detected in the SEI as indicated by B─O (191.4 eV), and B─F (194.3 eV) signals (Figure S20, Supporting Information). Fe 2p XPS spectrum (Figure S21, Supporting Information) showed deconvoluted peaks at 708.3 (2p_3/2_) and 716.3 eV (2p_1/2_) for Fe^0^, 711.8 (2p_3/2_) and 720.8 eV (2p_1/2_) for Fe^2+^.^[^ ^15^ ^]^ TOF‐SIMS depth profiling provided a more direct visualization of the composition of the SEIs. The abundant organics (C_3_^−^, CHO_2_^−^, C_4_^−^, C_4_H^−^, C_2_HO^−^) alongside Fe^−^, FeCl_2_^−^, and CaCl_2_^−^ fragments on FeCl_2_‐treated Ca were detected (Figure 4d; Figure S22, Supporting Information). The weaker signals of CO_3_^−^, CaF_2_^−^, and F^−^ confirmed much lower content of CaCO_3_/CaF_2_ species in the SEI of FeCl_2_‐treated Ca, in sharp contrast to that of pristine Ca (Figure 4d,e; Figure S23, Supporting Information). The organic species are also substantiated by the remarkable absorbance bands at 1062 cm^−1^ (C─O), 1660 cm^−1^ (C═O), 1509 cm^−1^ (C─C), and 1395 cm^−1^ (C─N) in FT‐IR spectra (Figure S24, Supporting Information). The above discussion reveals that FeCl_2_‐treated Ca develops organic‐rich SEIs containing fragmented Fe particles, Fe/Ca salts, and C─N species, facilitating Ca^2+^ diffusion kinetics while suppressing electrolyte decomposition.

### 2.4 Device Demonstration of the Ca‐Metal Anodes

**Figure 5:**
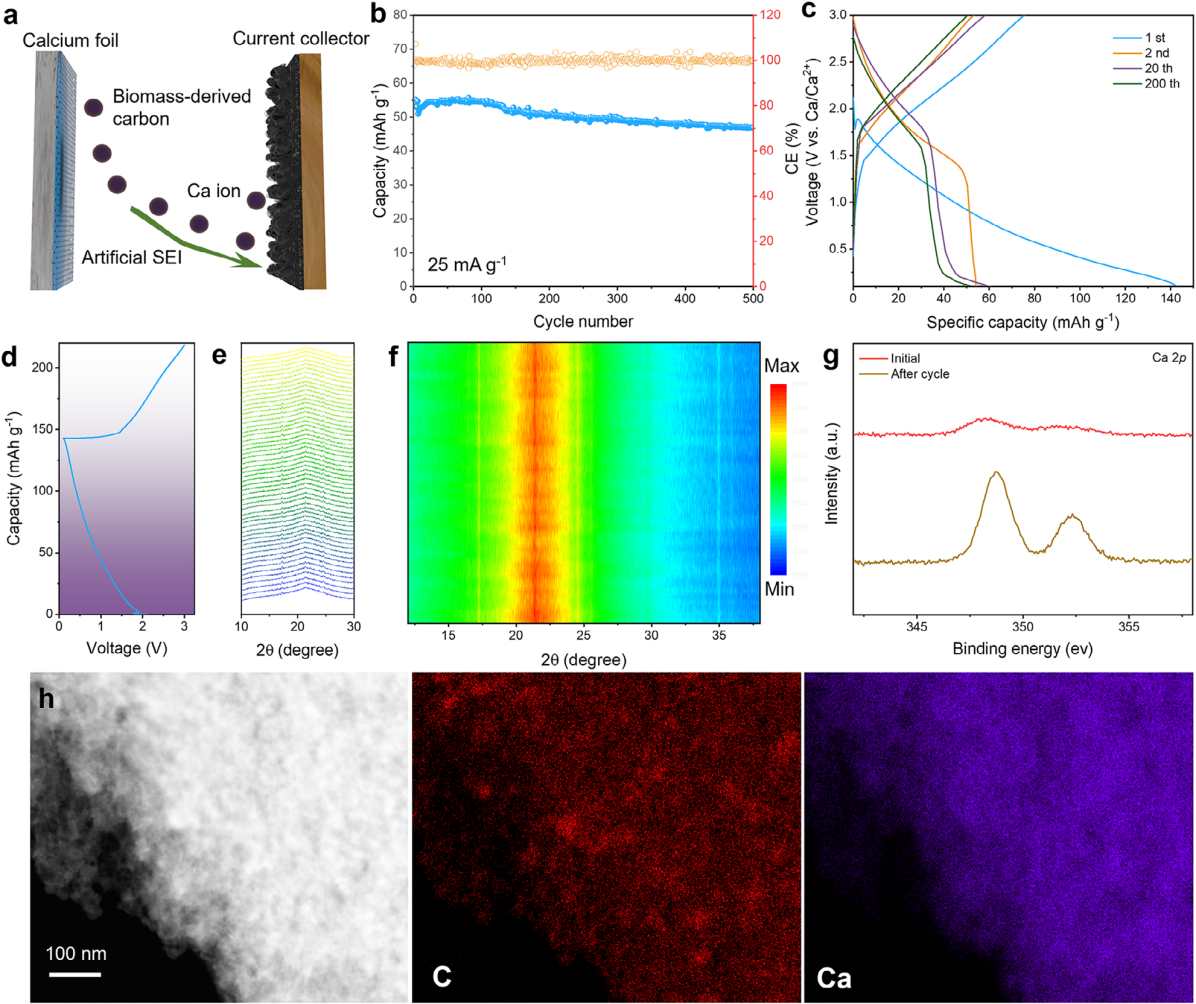
Electrochemical performance of biomass‐derived carbon || FeCl_2_‐treated Ca batteries. a) Working principle diagram. b) Long‐term cycling performance and c) the initial charge‐discharge profiles. Energy storage mechanism of bio‐mass derived carbon cathodes revealed by d–f) operando XRD patterns and the corresponding contour color mapping image, g) local Ca 2p XPS at full charge/discharge stages, and (e) STEM‐EDS mapping images after discharge.

A freestanding biomass‐derived carbon membrane was used as the cathode to demonstrate the applicability of the Ca‐metal anodes for full Ca‐metal batteries (**Figure** **5**a), as biomass derivatives feature a variety of advantages of excellent applicability, sustainability, and compatibility with fast kinetics.^[^ ^23^ ^]^ The biomass‐derived carbon exhibits a characteristic (002) plane diffraction peak at ≈21° (Figure S25, Supporting Information). The SEM image (Figure S26, Supporting Information) reveals a fibrous framework with abundant nanopores at the micron scale. The corresponding EDS indicates that the fibers are composed of C and O elements, with no detectable impurities.

At a current rate of 25 mA g^−1^, the Ca metal battery delivered a reversible capacity of 55 mAh g^−1^, which stably lasted for 500 cycles with a capacity retention of 85.4% (Figure 5b), being the longest cycle life for pure calcium salt ester‐based Ca‐metal batteries so far. Notably, atypical discharge behavior emerges with Ca^2^⁺ insertion occurring above 1 V (Figure 5c), distinguishing it from conventional lithium‐ion battery mechanisms.^[^ ^24^ ^]^ Ex‐situ Raman spectroscopy (Figure S27, Supporting Information) reveals that the intensity ratio of the disorder band to graphite band (I_D_/I_G_) decreases from 1.28 to 1.07 during discharge, indicating defect site adsorption of Ca^2^⁺ ions and consequent attenuation of defect‐related signals. The ratio subsequently increases during charging, demonstrating excellent reversibility of the calcium storage process. This anomalous voltage profile suggests a non‐classical storage mechanism where Ca^2+^ ions preferentially engage in surface adsorption within carbon nanopores rather than following traditional graphite intercalation pathways.^[^ ^25^ ^]^ The synergistic combination of hierarchical porosity and oxygen‐functionalized surfaces likely facilitates reduced activation energy for Ca^2^⁺ migration. Operando XRD analysis (Figure 5d–f), revealing absent stage transitions, further corroborates that the storage process is dominated by nanopore‐confined surface adsorption rather than crystalline phase intercalation. The XPS results (Figure 5g) confirm the successful insertion of calcium into the carbon cathode matrix, which aligns with the elemental distribution patterns observed in TEM and the corresponding EDS results (Figure 5h).

## 3 Conclusion

In summary, we developed a simple reaction ion exchange method to construct proper interphase species for Ca‐metal anodes reversible in the Ca(BF_4_)_2_ PC/DMC electrolyte. Notably, the interphase features abundant Fe nanoparticles encapsulated by an organic layer. During cycling, this interphase evolves into a compact SEI. The SEI not only accelerates Ca reaction kinetics but also mitigates anion decomposition at the Ca anode. Moreover, as a proof of concept, the corresponding Ca‐metal batteries demonstrated stable cycling for over 500 cycles. These results highlight the critical role of engineered interphases in mitigating parasitic reactions. The dense artificial SEI effectively isolates the metallic anode from corrosive electrolyte components, preserving structural stability and maintaining ionic conduction pathways. This study advances the understanding of Ca‐metal anode behavior and establishes a scalable interphase design strategy for Ca‐metal batteries using ester‐based electrolytes.

## Conflict of Interest

The authors declare no conflict of interest.

## Author Contributions

X.H. wrote the original draft, provided resources, and was responsible for methodology, investigation, formal analysis, and data curation. J.W., Q.C., Y.H., H.L., and F.T. contributed to writing, reviewing, and editing the manuscript. H.S. and C.W. participated in writing, reviewing, and editing, and were also responsible for validation, supervision, funding acquisition, and conceptualization.

## Supporting information



Supporting Information

## Data Availability

The data that support the findings of this study are available from the corresponding author upon reasonable request.
