# Supplementary material for: Anion‐Blocking and Multipath‐Conducting Interfaces Enable Long‐Life Room‐Temperature Ester‐Based Ca‐Metal Batteries
Source: Adv Sci (Weinh). 2025 Sep 12;12(45):e12339. doi: 10.1002/advs.202512339 (PMC12677698; doi:10.1002/advs.202512339)
Supplement: Supplementary file 1 — Supporting Information [file ADVS-12-e12339-s001.docx]

Supporting Information for

**Anion-Blocking and Multipath-Conducting Interfaces Enable Long-Life Room-Temperature Ester-Based Ca-Metal Batteries**

*Xuedong He*, *Jiarui Wang*, *Qingyang Cao*, *Yaohua Huang*, *Hongqing Li*, *Fei Tian*, *Huawei Song*, * *and Chengxin Wang* *

Dr. Xuedong He, Jiarui Wang, Qingyang Cao, Yaohua Huang, Dr. Hongqing Li, Prof. Fei Tian, Prof. Huawei Song, Prof. Chengxin Wang

State Key Laboratory of Optoelectronic Materials and Technologies, School of Materials Science and Engineering, Sun Yat-Sen (Zhongshan) University, Guangzhou 510275, People’s Republic of China

E-mail: songhw5@mail.sysu.edu.cn (Prof. Huawei Song), wchengx@mail.sysu.edu.cn (Prof. Chengxin Wang)

**Experimental Section**

*Preparation of Ca-Metal Anodes with Artificial Interphases*: Ferrous chloride (FeCl_2_, Aladdin, 98%), N, N-dimethylformamide (DMF, Aladdin, 99.9%), calcium tetrafluoroborate hydrate (Ca(BF_4_)_2_, Alfa Aesar, Ca 12% min), propylene carbonate (PC, Aladdin, 99%), dimethyl carbonate (DMC, Aladdin, 99%), Ca granules (Sigma Aldrich, 99.9%), and acetylene black carbon (Shenzhen Kejing) were used without further purification. Commercial Ca granules were pressed into pieces of Ca foil. Pristine Ca anodes were obtained by polishing the foil and tailoring to appropriate size. The Ca-metal anodes with multipath conduction interfaces were obtained by drop-casting 40 μL 0.5 M FeCl_2_ DMF solution onto the pristine Ca-metal anode, and then reacted for 30 min. The above reaction procedures were conducted in an Ar-filled glove box (<0.1 ppm of O_2_, <0.1 ppm of H_2_O).

*Preparation of Biomass-Derived Carbon Membrane Cathode*: The freestanding biomass-derived carbon membrane was prepared by vacuum pyrolysis of cellulose paper at 800 °C for 2 h in a tube furnace with a heating rate of 10 °C/min, and then naturally cooled to room temperature.

*Materials Characterization*: X-ray diffraction (XRD) was carried out using a RIGAKU (D-MAX 2200 VPC) instrument with Cu Kα radiation (λ = 0.15406 nm), collecting data within a range of 2θ =10-80° with a step size of 0.02°. Scanning electron microscopy (SEM) and transmission electron microscopy (TEM) images were acquired using a Nova Nano SEM 200 and FEI Tecnai G2 F30, respectively. X-ray photoelectron spectroscopy (XPS) was performed utilizing a spectrometer from Kratos Axis Ultra DLD, employing Mono Al Kα radiation at 120 W power (8 mA, 15 kV). Fourier Transform infrared spectroscopy (FT-IR) was carried out using a Vertex70-Hyperion3000. The time of flight secondary ion mass spectrometry (TOF-SIMS) was performed in a negative-ion mode by IONTOF GmbH, Germany.

*Electrochemical Measurements*: The assembly of the battery (2032 type) was conducted in a glove box filled with Ar. The Ca plating/stripping reversibility was evaluated through the polarization potential change at a constant current density. Ca-metal electrodes were used as both the anode and cathode, glass microfiber filters (Whatman) were used as the separator, 0.5 M Ca(BF_4_)_2_ PC/DMC (v/v = 1:1) (4 Å molecular sieves were added to remove residual water in the electrolyte for 48 hours) was used as the electrolyte. DMF-treated Ca and pristine Ca electrodes were used for comparison. The battery was tested on a Neware battery testing system (Neware, CT-4008Tn). A freestanding biomass-derived carbon membrane was used as the cathode for Ca-metal full batteries. The three-electrode system was performed with an Ag electrode containing 0.1 M AgPF_6_ dissolved in PC/DMC as the reference electrode. The electrochemical impedance spectroscopy (EIS) and cyclic voltammetry (CV) were carried out on an electrochemical workstation (Donghua, DH7006B), employing a frequency range from 100 kHz to 0.01 Hz and an amplitude of 5 mV for the EIS and a scan rate of 1 mV s^−1^ for the CV.


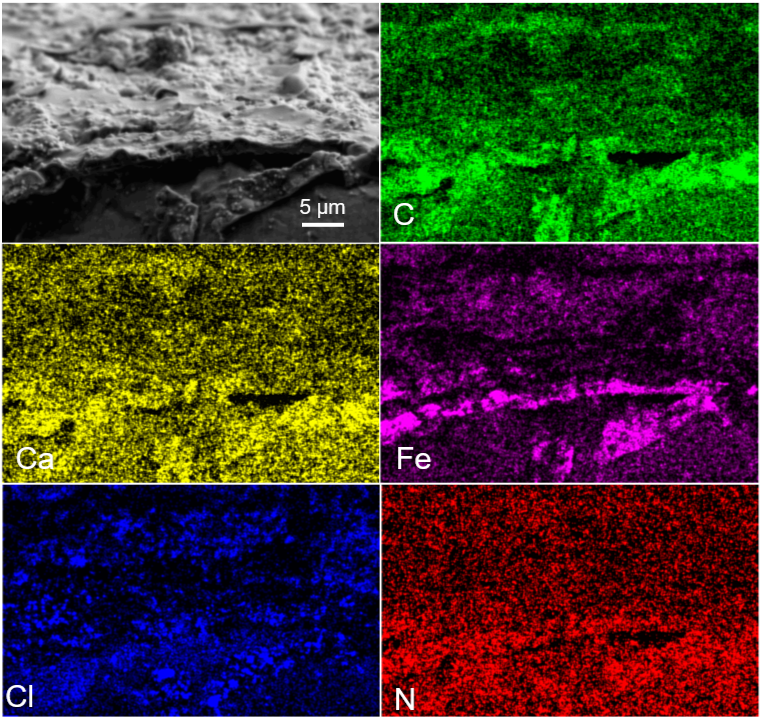


**Figure S1**. Cross-sectional SEM, and the corresponding EDS mapping images of FeCl_2_-treated Ca electrodes before cycling.


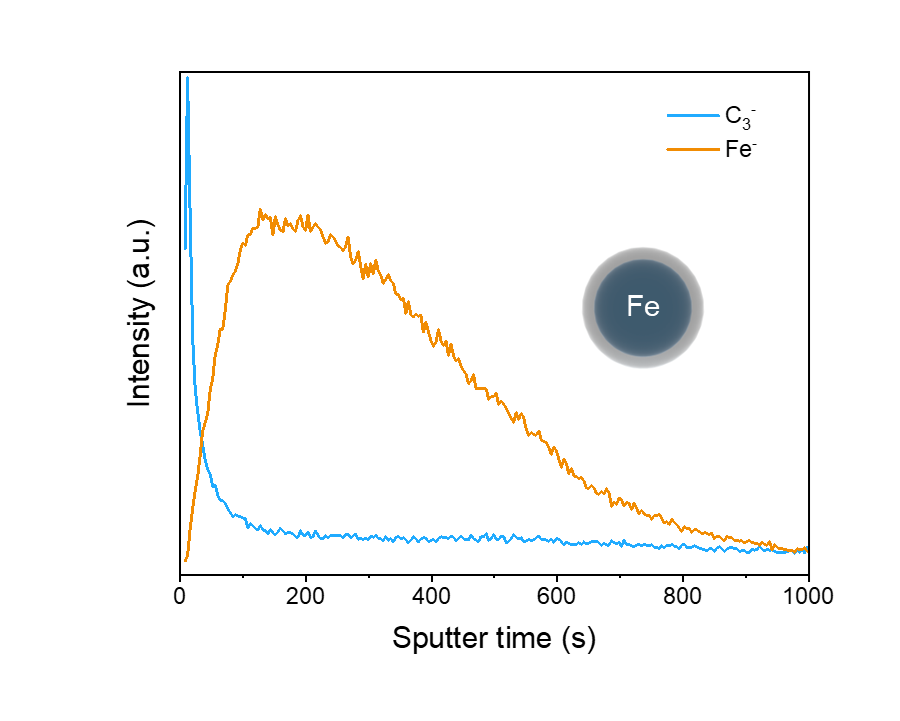


**Figure S2**. TOF-SIMS depth profiling of FeCl_2_-treated Ca electrodes before cycling.

Count intensity of C_3_^-^ and Fe^-^ fragments versus sputter time.

**
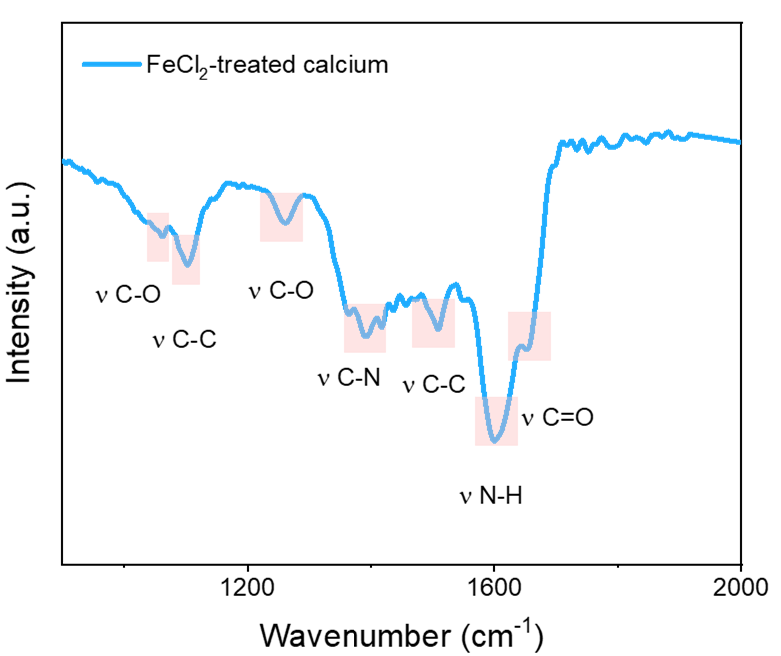
**

**Figure S3**. FT-IR spectrum of FeCl_2_-treated Ca electrodes before cycling.


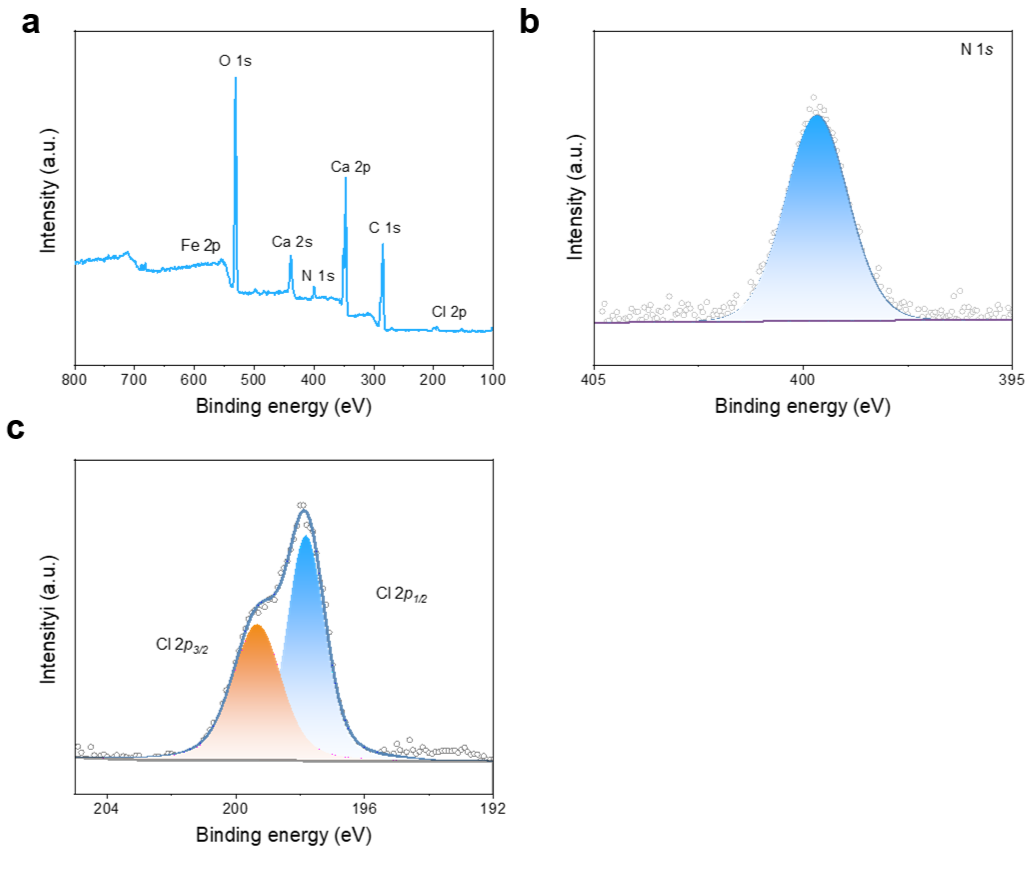


**Figure S4**. XPS spectra of FeCl_2_-treated Ca electrodes before cycling. (a) Survey spectrum, deconvoluted local (b) N 1s, and (c) Cl 2p spectra.


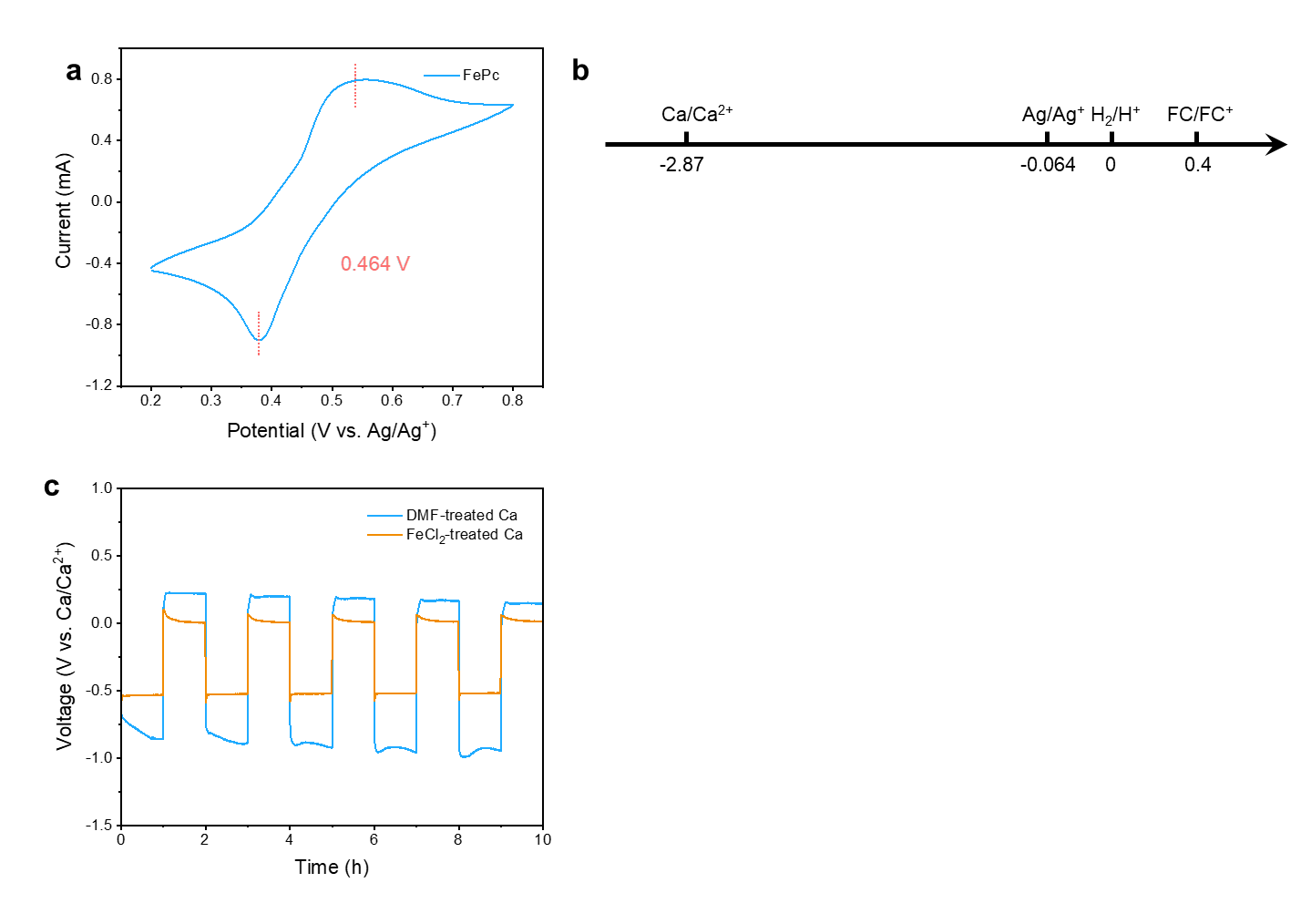


**Figure S5**. (a) CV curve of 5 mM ferrocene dissolved in electrolyte at a scan rate of 10 mV s^-1^. (b) The Ag/Ag^+^ potential determined by Swagelok-based three-electrode. (c) Calibrated plating/stripping curves of three-electrode cell configuration at 0.02 mA cm^-2^ for 1 h.


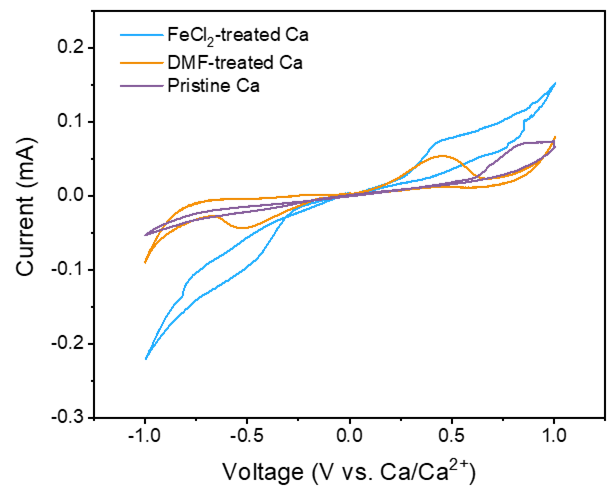


**Figure S6**. CV curves for symmetric batteries of different Ca-metal electrodes tested at 1 mV s^−1^.


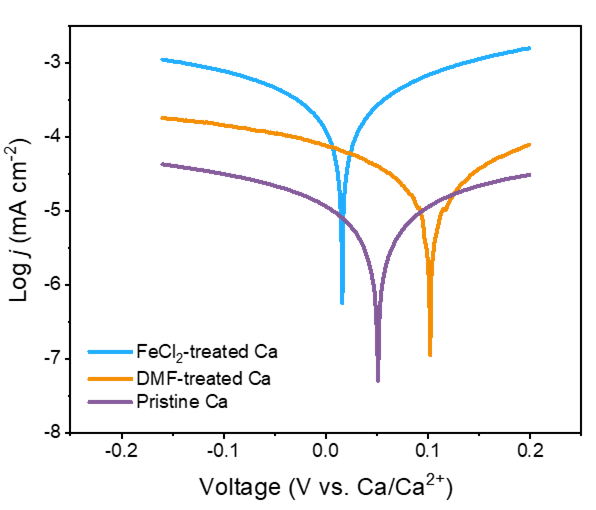


**Figure S7**. Tafel plots of FeCl_2_-treated Ca, DMF-treated Ca, and pristine Ca.


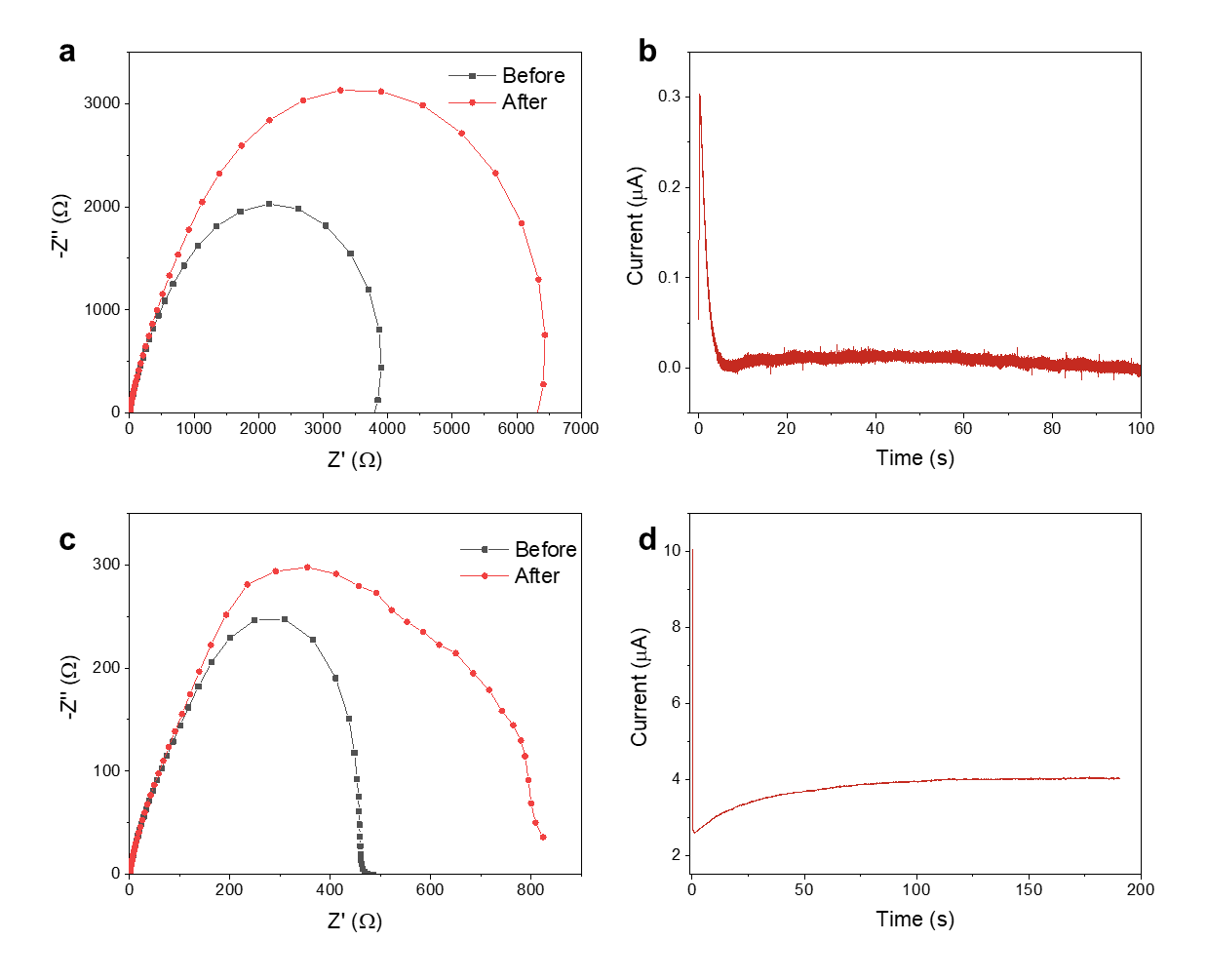


**Figure S8.** Ca^2+^ transference number for EIS before and after polarization of (a) Pristine Ca, and (c) FeCl_2_-treated Ca, Chronoamperometry test at 20 mV of (b) Pristine Ca, and (d) FeCl_2_-treated Ca.

For transfer number testing, Ca symmetric cells were assembled. The transfer number of the cell was calculated using the following equation:

$$t=\frac{I_{s}(\Delta V-I_{0}R_{0})}{I_{0}(\Delta V-I_{s}R_{s})}$$

where$\Delta V$ is the constant polarization voltage applied, $I_{0}$ and $R_{0}$ are the initial current and resistance, $I_{s}$ and $R_{s}$ are the steady-state current and resistance, respectively.


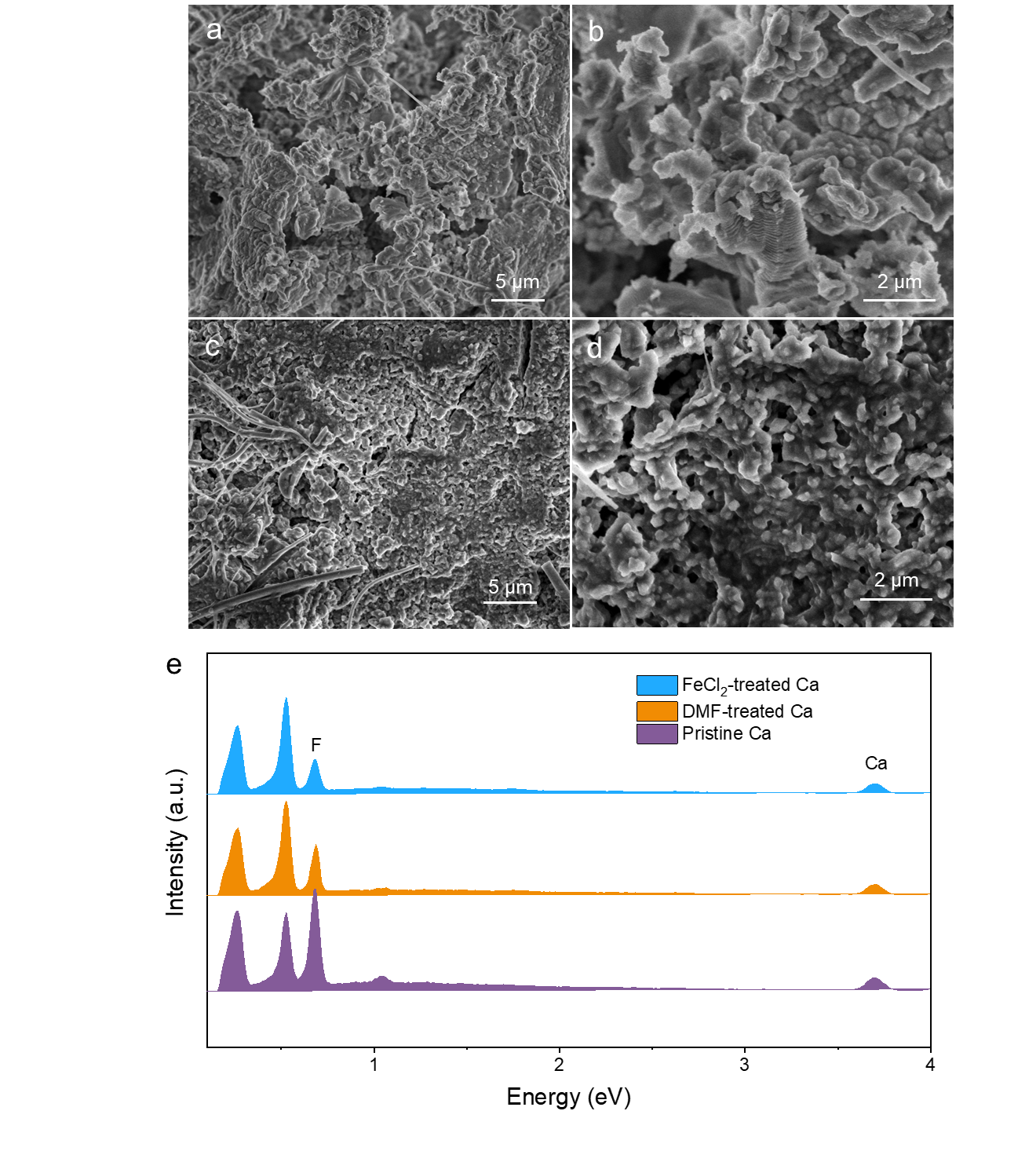


**Figure S9**. SEM images of (a, b) pristine Ca, (c, d) DMF-treated Ca after plating for 3 mAh cm^-2^, and corresponding EDS.


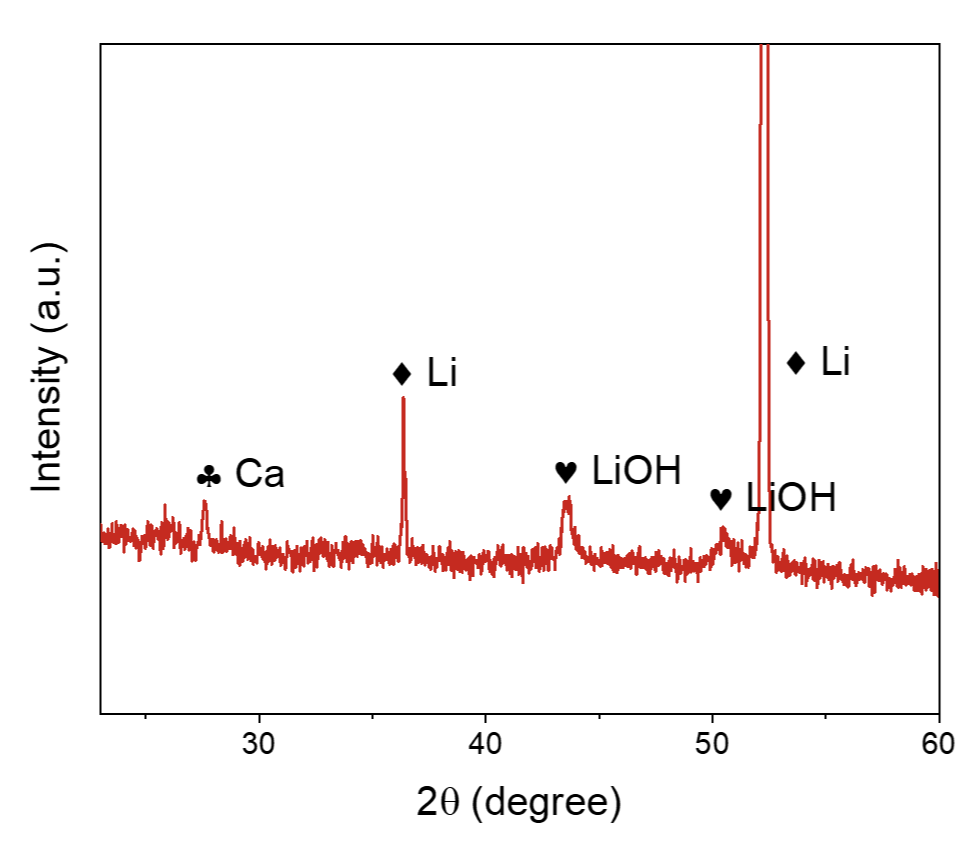


**Figure S10**. XRD pattern of Ca deposition on FeCl_2_-treated Li foil.


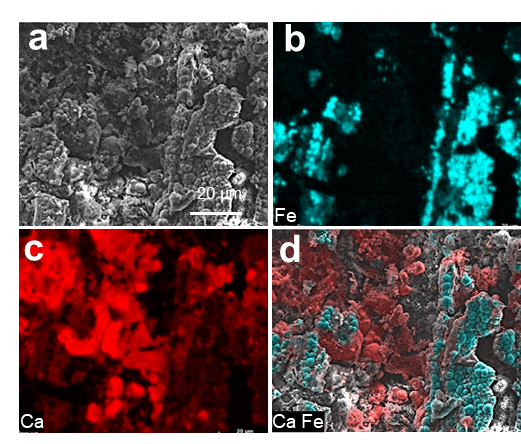


**Figure S11**. (a) SEM image of FeCl_2_-treated Li foil after Ca plating 1 mA h cm^-2^, and corresponding (b) Fe, (c) Ca EDS mapping. (d) SEM and Fe Ca EDS mapping overlay.


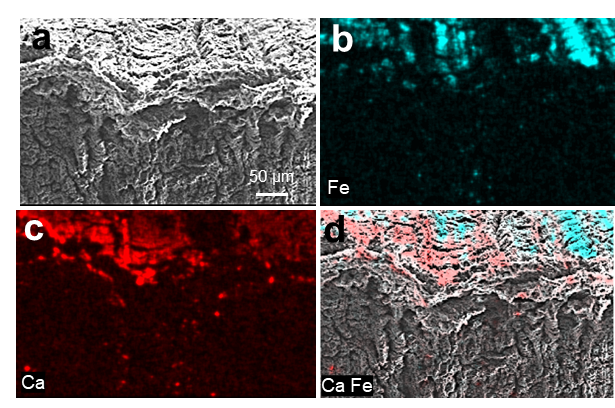


**Figure S12**. (a) Cross-sectional SEM image of FeCl_2_-treated Li foil after Ca plating 1 mA h cm^-2^, and corresponding (b) Fe, (c) Ca EDS mapping. (d) SEM and Fe Ca EDS mapping overlay.


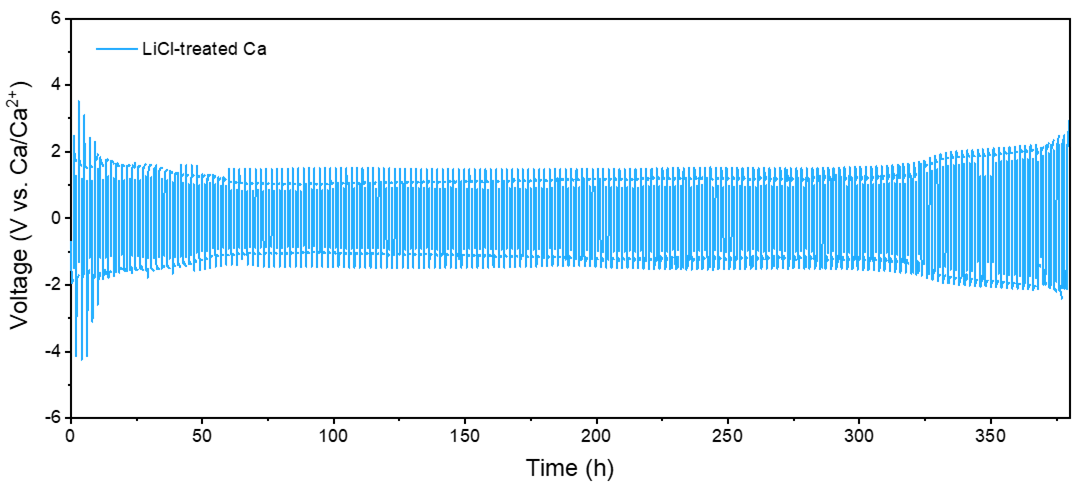


**Figure S13**. Voltage-time profiles for cycling stability of LiCl-treated Ca electrodes at 0.02 mA cm^−2^.


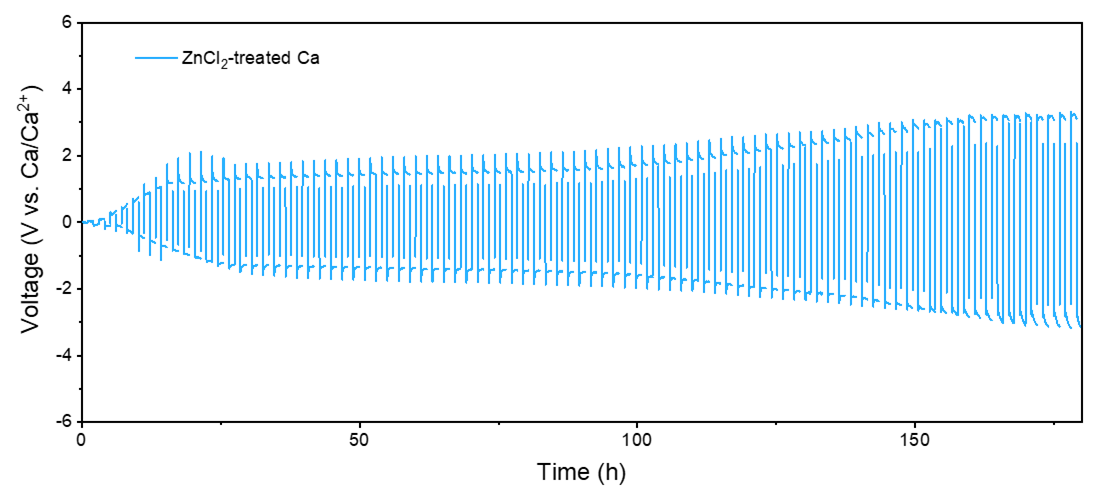


**Figure S14**. Voltage-time profiles for cycling stability of ZnCl_2_-treated Ca electrodes at 0.02 mA cm^−2^.


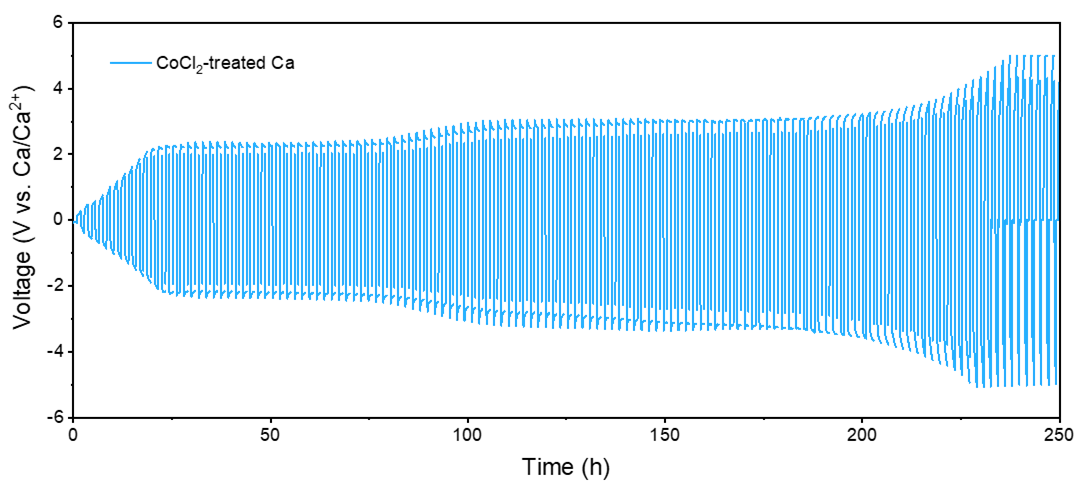


**Figure S15**. Voltage-time profiles for cycling stability of CoCl_2_-treated Ca electrodes at 0.02 mA cm^−2^.


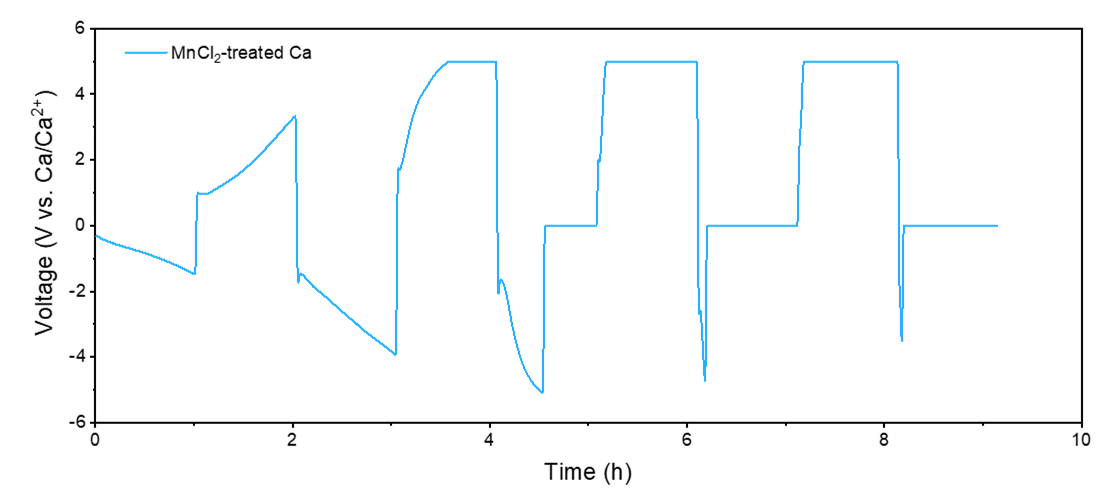


**Figure S16**. Voltage-time profiles for cycling stability of MnCl_2_-treated Ca electrodes at 0.02 mA cm^−2^.


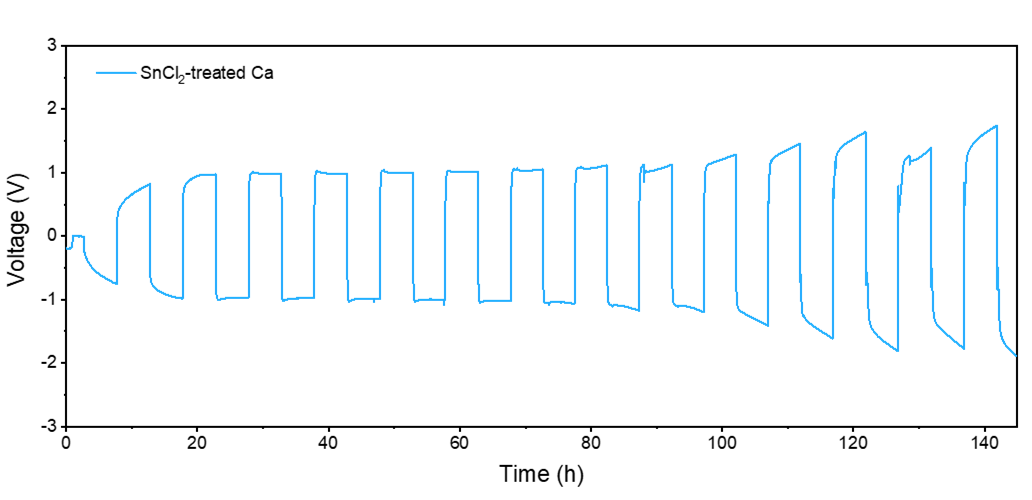


**Figure S17**. Voltage-time profiles for cycling stability of SnCl_2_-treated Ca electrodes at 0.02 mA cm^−2^.


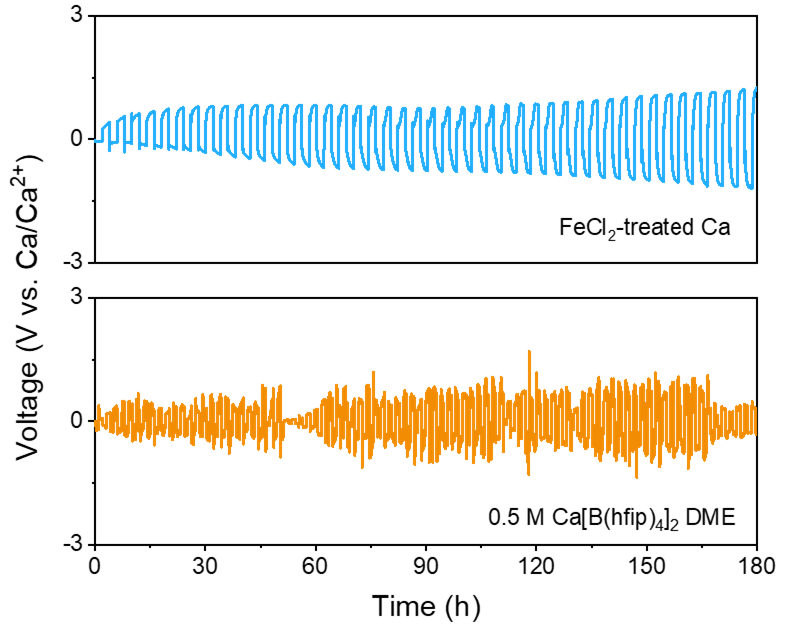


**Figure S18**. Voltage-time profiles for cycling stability of FeCl_2_-treated Ca electrodes at 0.1 mA cm^−2^. (The figure above shows the performance of the artificial interface after modification proposed in this paper, and the figure below shows the performance of the electrolyte reported in the literature, *Energy Environ. Sci.*, 2019, 12(12): 3496-3501.)


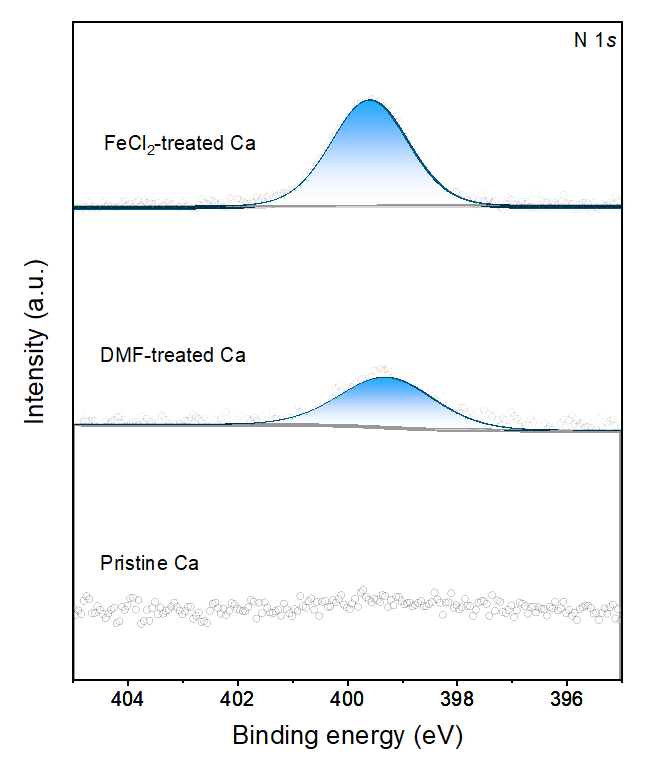


**Figure S19.** Deconvoluted local N 1s XPS spectra of different Ca-metal electrodes after cycling.


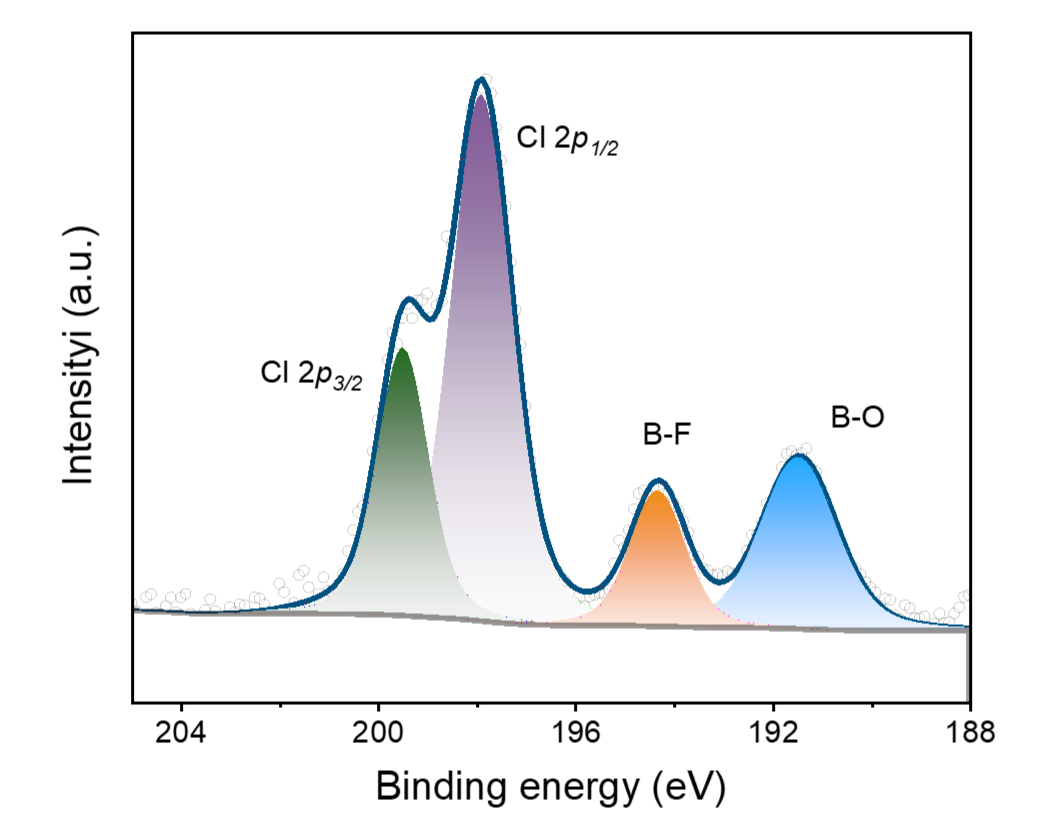


**Figure S20.** Deconvoluted local B 1s and Cl 2p XPS spectra FeCl_2_-treated Ca after cycling.


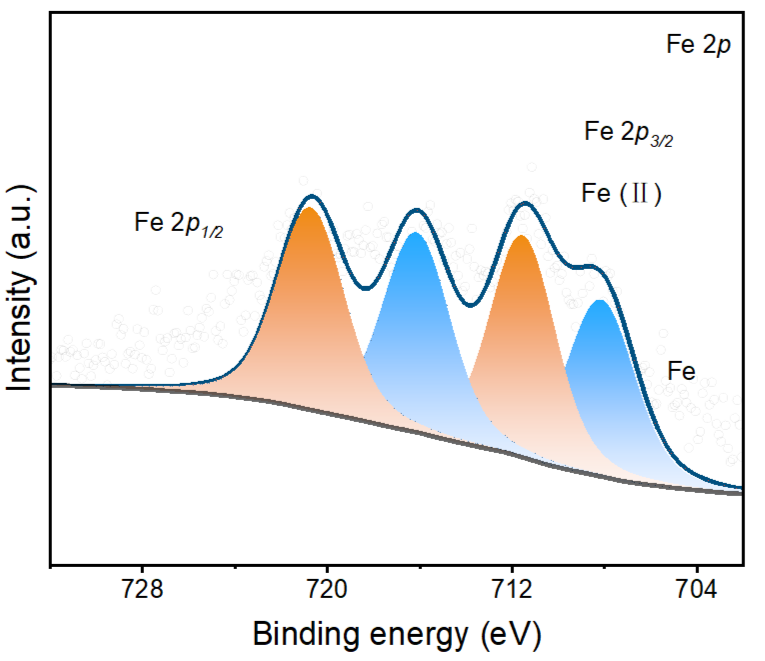


**Figure S21.** Deconvoluted local Fe 2p XPS spectra FeCl_2_-treated Ca after cycling.

**
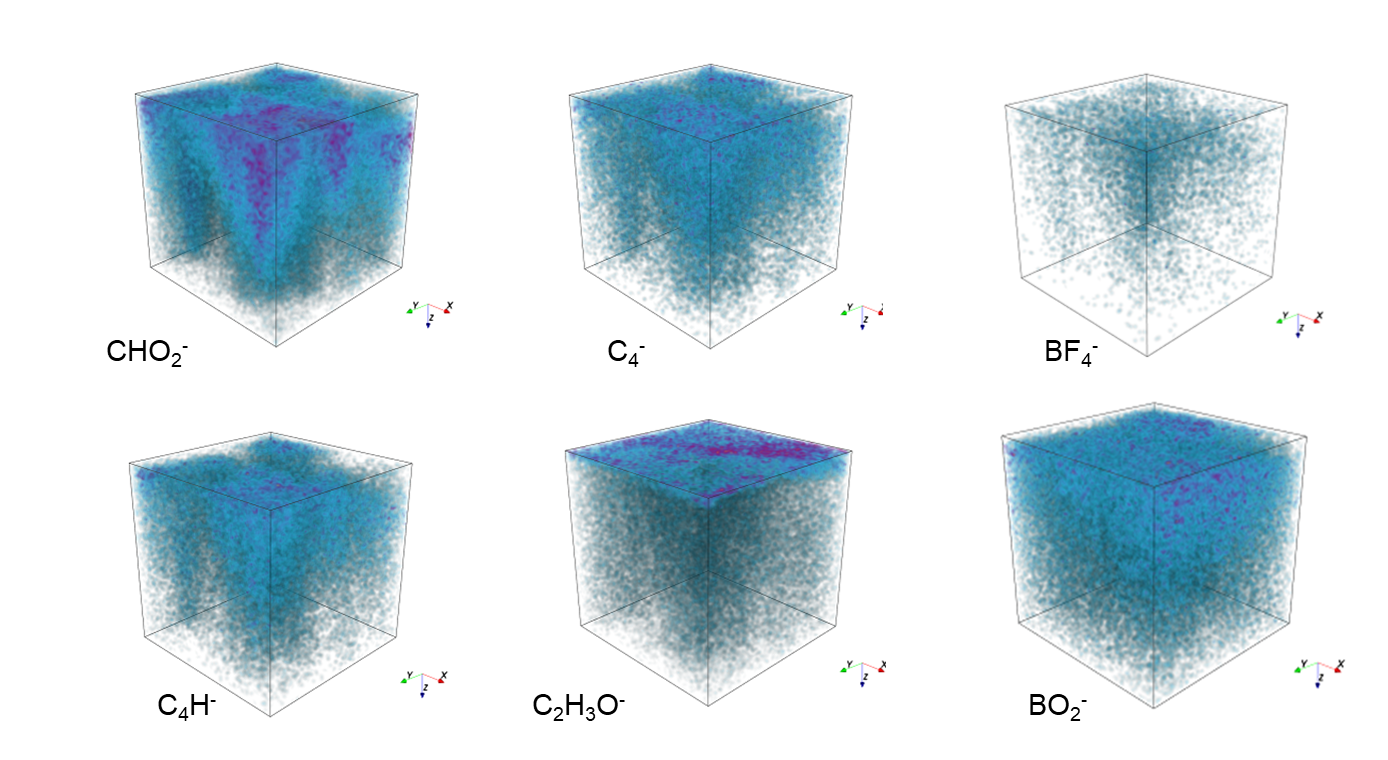
**

**Figure S22**. TOF-SIMS 3D render overlay images of FeCl_2_-treated Ca.

**
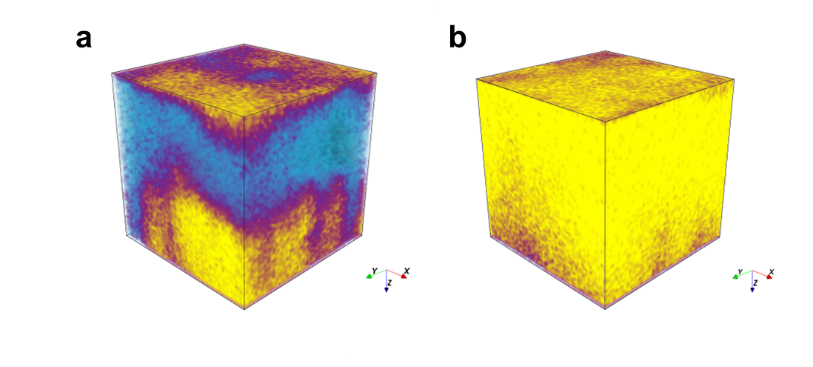
**

**Figure S23.** F^-^ signal of TOF-SIMS 3D render overlay images of (a) FeCl_2_-treated Ca, and (b) pristine Ca.


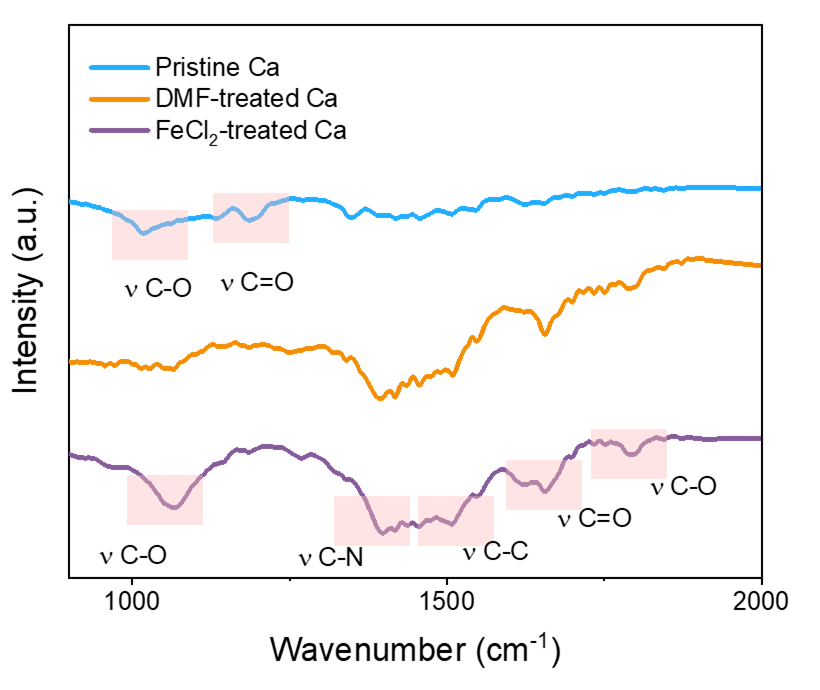


**Figure S24.** FT-IR spectra of pristine Ca, DMF-treated Ca, and FeCl_2_-treated Ca after 10 plating/stripping cycles.


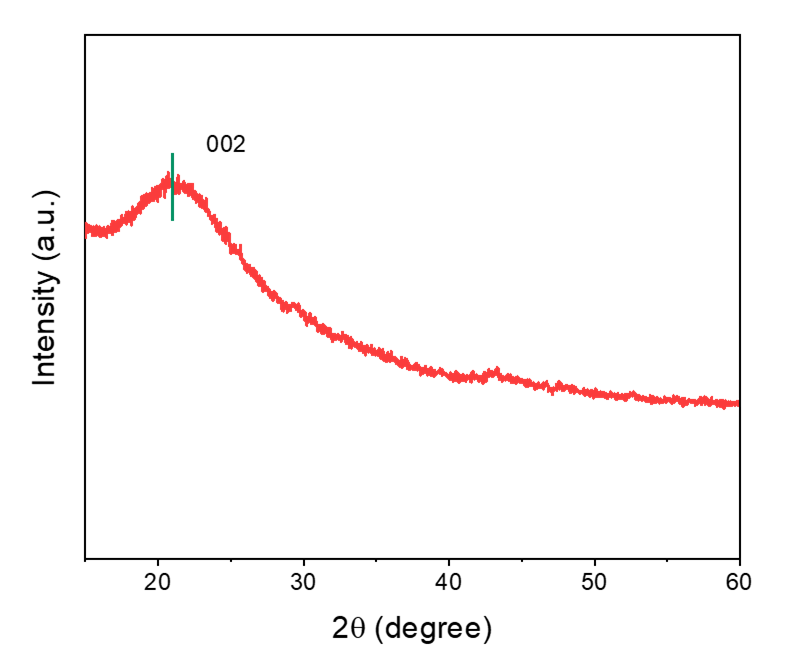


**Figure S25.** XRD pattern of the biomass-derived carbon**.**

**
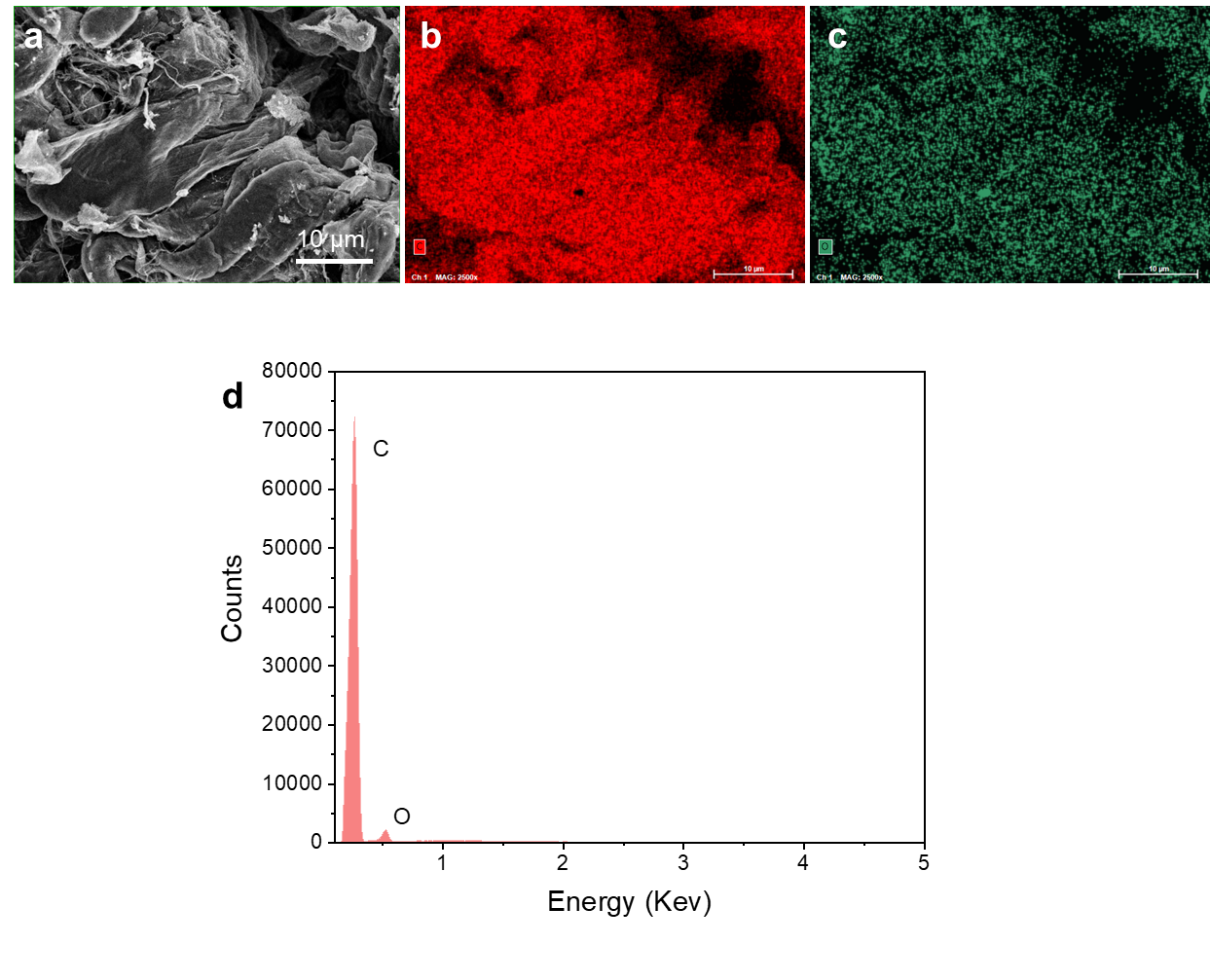
**

**Figure S26.** (a) SEM image and corresponding (b, c) EDS mapping images, and (d) EDS spectrum.


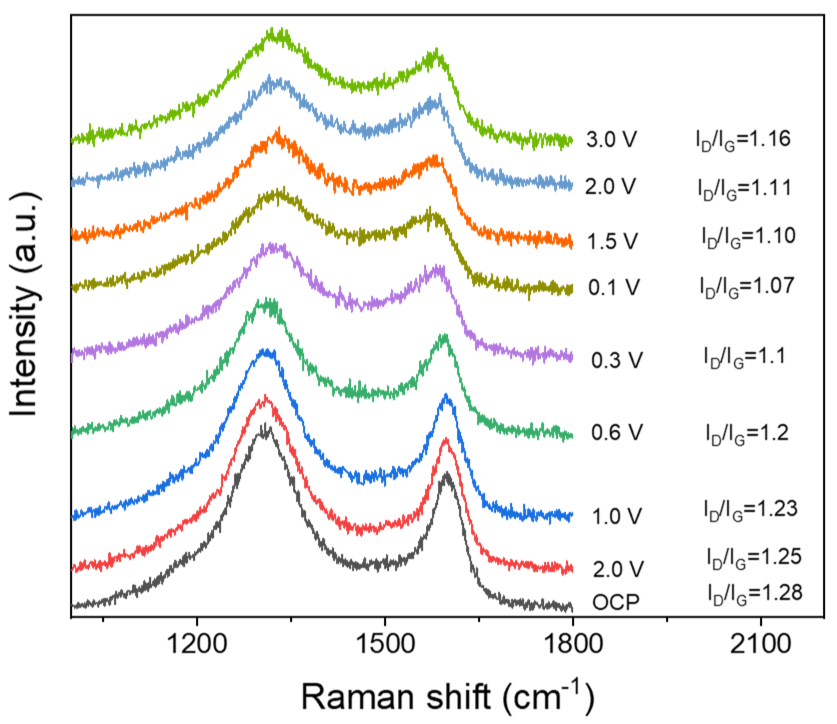


**Figure S27.** Ex-situ Raman spectroscopy of bio-mass derived carbon at full charge/discharge stages

**Table S1**. Summary of Ca reversibility in previous reports.

| **Anode** | **Electrolyte** | **Current density** | **Time** | **Reference** |
| --- | --- | --- | --- | --- |
| Ca | 0.5 M Ca(BF_4_)_2_ PC/DMC | 0.02 | 950 | This Work |
| Ca | 1 M NaPF_6_ EC/DMC/EMC | 0.1 | 200 | Advanced Materials, 2021, 33(2): 2006141. |
| Ca | 0.1 M Ca(BF_4_)_2_-0.9 M NaPF_6_ | 0.025 | 400 | Advanced Functional Materials, 2022, 32(21): 2200004. |
| Ca | Ca-soaked electrolyte | 0.02 | 500 | Advanced Energy Materials, 2021, 11(10): 2003685. |
| Ca | Ca(TFSI)_2_ EC/DEC | 0.005 | 420 | Chemical Engineering Journal, 2024: 157793. |
| AHSEL-Ca | 1 M KPF_6_ EC/DMC/EMC | 0.1 | 300 | Energy & Environmental Materials, 2023, 6(2): e12325. |
| Ca | 0.5 M Ca(BH_4_)_2_+0.1 M LiBH4/THF | 0.1 | 200 | ACS Applied Materials & Interfaces, 2022, 14(49): 54616-54622. |
| Ca | 0.45 M Ca(BF_4_) _2_ in EC:PC @100℃ | 0.05 | 92 | Nature Materials, 2016, 15(2): 169-172. |
| Ca-20h-CV | 0.25 M Ca[B(hfip)_4_]_2_/DME | 1 | 180 | Energy & Environmental Science, 2024, 17(18): 6548-6558. |
| Ca | GTHFB | 0.05 | 100 | Angewandte Chemie International Edition, e202413416. |
| Ca | Ca(BH(pftb)_3_)_2_ DME | 1.5 | 90 | Nature Energy, 2024, 9(3): 285-297. |
| Ca | Ca(BF_4_)_2_ ionic liquids | 0.55 | 80 | ACS Applied Energy Materials, 2020, 3(3): 2310-2314. |
| Ca | Ca(TFSI)_2_ DMAc | 0.02 | 100 | Angewandte Chemie, 2022, 134(50): e202214796. |
| Au | Ca(BH_4_)_2_ (THF) | 1 | 100 | Nature materials, 2018, 17(1): 16-20. |
| Ca | Ca(B(Ohfip)_4_)_2_ DME | 0.2 | 100 | ACS Energy Letters, 2019, 4(9): 2271-2276. |
| Ca | 0.4 m Ca(BH_4_)_2_ and 0.4 m LiBH_4_ in THF | 1 | 100 | Angewandte Chemie International Edition, 2020, 59(31): 12689-12693. |
